# Reversion of breast epithelial polarity alterations caused by obesity

**DOI:** 10.1038/s41523-023-00539-w

**Published:** 2023-05-09

**Authors:** Julia Holmes, Mohamed Gaber, Mónica Z. Jenks, Adam Wilson, Tucker Loy, Cassandra Lepetit, Mara Z. Vitolins, Brittney-Shea Herbert, Katherine L. Cook, Pierre-Alexandre Vidi

**Affiliations:** 1 Department of Cancer Biology, Wake Forest University School of Medicine, Winston-Salem, NC 27157 USA; 2 Department of Surgery, Wake Forest University School of Medicine, Winston-Salem, NC 27157 USA; 3 Institut de Cancérologie de l’Ouest, Angers, 49055 France; 4 Department of Epidemiology and Prevention, Wake Forest University School of Medicine, Winston-Salem, NC 27157 USA; 5 Department of Medical & Molecular Genetics, IU School of Medicine, Indianapolis, IN 46202 USA; 6 Atrium Health Wake Forest Baptist Comprehensive Cancer Center, Winston-Salem, NC USA

**Keywords:** Cell polarity, Predictive markers

## Abstract

Molecular links between breast cancer risk factors and pro-oncogenic tissue alterations are poorly understood. The goal of this study was to characterize the impact of overweight and obesity on tissue markers of risk, using normal breast biopsies, a mouse model of diet-induced obesity, and cultured breast acini. Proliferation and alteration of epithelial polarity, both necessary for tumor initiation, were quantified by immunostaining. High BMI (>30) and elevated leptin were associated with compromised epithelial polarity whereas overweight was associated with a modest increase in proliferation in human and mice mammary glands. Human serum with unfavorable adipokine levels altered epithelial polarization of cultured acini, recapitulating the effect of leptin. Weight loss in mice led to metabolic improvements and restored epithelial polarity. In acini cultures, alteration of epithelial polarity was prevented by antioxidants and could be reverted by normalizing culture conditions. This study shows that obesity and/or dietary factors modulate tissue markers of risk. It provides a framework to set target values for metabolic improvements and to assess the efficacy of interventional studies aimed at reducing breast cancer risk.

## Introduction

Breast cancer incidence has been rising globally over the last three decades^1^. Beyond therapeutic developments, greater emphasis needs to be placed on primary prevention to address this societal burden. Aging is a major risk factor for breast cancer. Yet increase in incidence has been higher in women under 50 than in older women, suggesting causes beyond the expansion in life expectancy. This increase in younger women also indicates that improved screening, which generally start at 50, is not the only explanation for increased breast cancer incidence. Rather, evolution in lifestyle and environmental exposures likely explain much of the increase in breast cancer risk. Some behavioral changes, in particular those related to reproductive patterns, cannot (and arguably should not) be reverted to pre-industrialization times, while other shifting behaviors, including excess energy intake, westernization of dietary patterns, and reduced physical activity are more amenable to interventions. It is estimated that >50% of breast cancers could be avoided with risk-reducing behaviors^2^.

Overweight and obesity increase breast cancer risk in postmenopausal^3^ and in high-risk pre-menopausal^4^ women. Increased estrogen production through the conversion of androgenic precursors in adipose tissue may account for 10–15% of the effect of overweight/obesity on post-menopausal breast cancer risk^5^. Other metabolic factors shifted in obesity are also independently associated with elevated risk. These include IGF-1^6^, insulin^7^, leptin^8–10^, and cholesterol^11^.

Our group and others previously documented that elevated leptin compromises breast tissue architecture by altering the localization and function of cell–cell junctions^12–14^. Cell-cell junctions comprise gap junctions, desmosomes, adherens junctions, and tight junctions (TJs). The latter are located above the other junction types, close to the lumen. They separate apical and basolateral membrane compartments, enabling directional transport towards the lumen. TJs, together with the other cell junctional complexes, are a key aspect of apical-basal polarity of breast luminal cells, which is essential for homeostasis of the epithelium^15–18^. Alteration of apical–basal polarity is one factor contributing to tumorigenesis. It occurs in early breast cancer stages and is often considered a necessary (albeit not sufficient) step for tumor initiation. Atypical ductal hyperplasia, a pre-malignant condition characterized by disorganized breast epithelium with multiple cell layers, is characterized by partial loss of apical–basal polarity. Moreover, the TJ-associated protein ZO-1 which regulates TJ assembly and anchors TJs to the cortical actin network, is lost, down-regulated, and/or mislocalized in a majority of breast cancers^19^. Alteration of apical-basal polarity leads to misregulation of proliferative and survival pathways, defective orientation of the mitotic spindle (necessary for cell multilayering), and the expansion of stem/progenitor cell populations—all hallmarks of cancer initiation. The goal of this study was to define the impact of overweight/obesity and of serum cytokines on epithelial polarity and proliferation of normal breast tissue, and to determine if alterations in these tissue markers of cancer risk are reversible. This knowledge is needed to identify interventions that have a measurable impact, at the tissue level, on breast cancer risk.

## Results

### High body mass index is associated with abnormal TJs in breast tissue

**Fig. 1:**
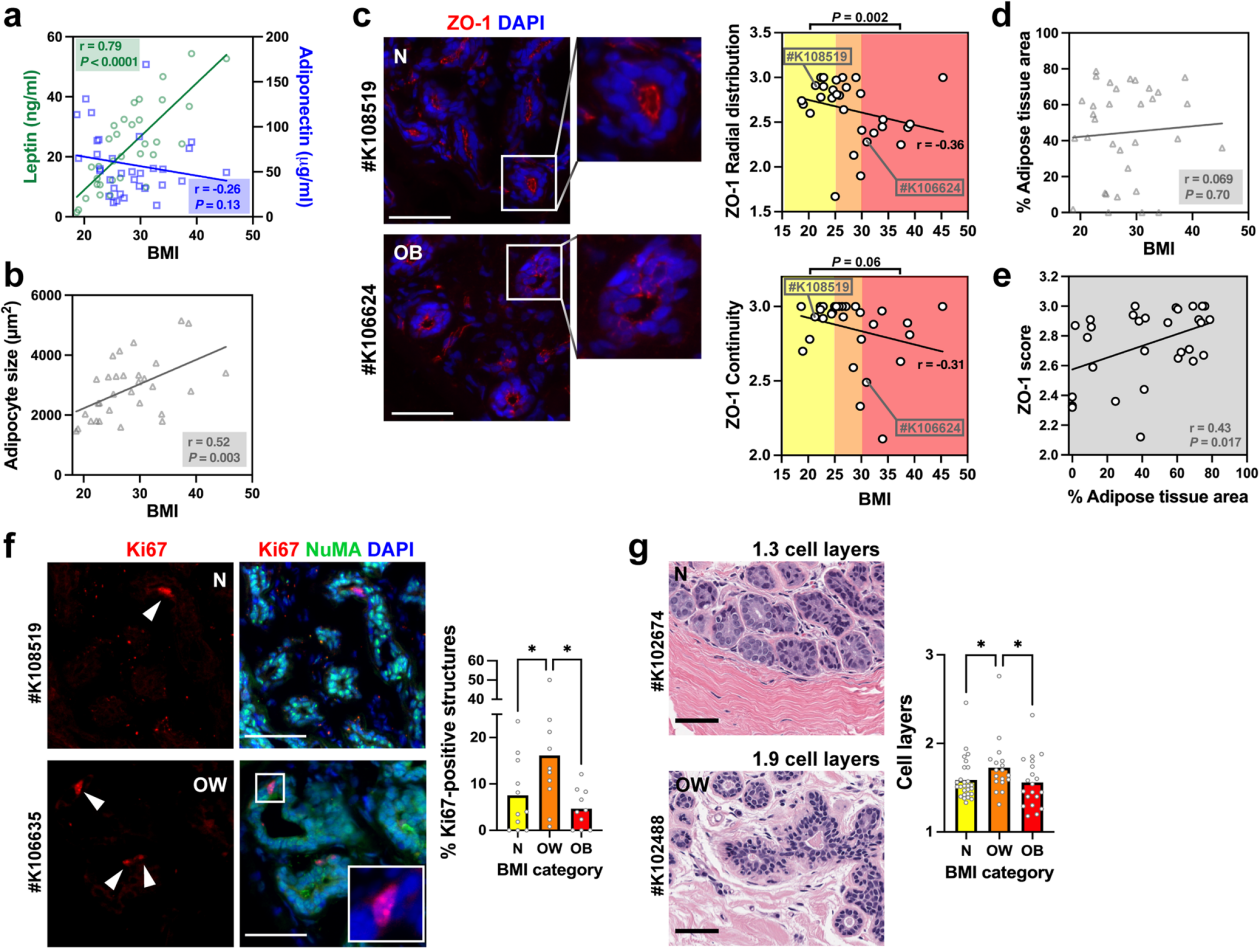
Effect of the body mass index on epithelial polarity and proliferation in normal breast tissues. **a** Leptin and adiponectin levels as a function of BMI in serum from women who donated breast tissue specimens to the Komen Tissue Bank. Spearman correlation coefficients (*r*) are indicated with corresponding *P* values. **b** Cross-sectional size of adipocytes in breast tissue sections from KTB donors. *r*, Pearson’s correlation coefficient. **c** ZO-1 radial distribution and continuity in breast tissue sections as a function of BMI. Microscopy images from two different donors are shown for illustration. Cell nuclei were stained with DAPI. Statistical comparisons (normal weight vs. obesity) with Mann Whitney test. **d** Relative proportion of adipose tissue as a function of BMI. *r*, Pearson’s coefficient. **e** ZO-1 score (average of continuity and radial distribution) as a function of the adipose content of the tissues. *r*, Pearson’s coefficient. **f** Quantification of epithelial structures (ducts, lobules) with ≥1 Ki67-positive cells according to BMI categories. Representative immunostaining images are shown, with arrowheads pointing to Ki67-positive nuclei. Antibodies against NuMA were used as staining control. *P < 0.05 (ANOVA and Fischer’s LSD test). **g** Average number of epithelial cell layers in breast epithelia from different BMI categories, assessed on H&E images. *P < 0.05 (Kruskal–Wallis and Dunn’s test). Averages are shown on bar graphs. Scale bars: 50 µm. N normal weight, OW overweight, OB obese. Each symbol on the graphs represents a KTB donors.

To determine if obesity affects cell–cell TJs, as suggested in our previous study^12^, we obtained normal breast tissue and matching serum samples from the Komen tissue bank, selecting donors with body mass indices (BMI) ranging from 18.7 to 45.3 (Table 1 and Supplementary Table 1). Donor age and race were balanced in the three BMI groups. We excluded hormone replacement therapy (HRT) users since HRT may increase breast cancer risk^20^. Smoking and alcohol use are additional possible confounding factors. Hence, samples from self-reported current smokers and alcohol consumers were also excluded. We first assessed a panel of adipokines, cytokines, and growth factors in the serum samples (Supplementary Table 2). As expected^12,21–25^, leptin levels strongly correlated with BMI (Fig. 1a). Weaker associations with BMI were also found for other soluble factors associated with obesity. Notably, adiponectin serum levels were lower in the overweight compared to the normal weight category, as reported previously^26^ (Fig. 1a and Supplementary Fig. 1a). We also observed slightly higher levels (not statistically significant) of insulin and IGF-1, as well as lower levels of IGFBP1 (but not IGFBP2) in the obese and overweight BMI categories compared to normal weight (Supplementary Fig. 1). The size of adipocytes positively correlated with BMI (Fig. 1b), further validating our study cohort.

**Table 1:** Characteristics of breast tissue donors by BMI categories.

Variable	Normal weight	Overweight	Obese	*P* value
*N*	11	11	11	
BMI	21.9 ± 1.9	27.1 ± 1.8	35.0 ± 4.6	<0.0001
Age	34.8 ± 10.1	28.1 ± 9.6	34.9 ± 11.4	0.22
Race (% W/AA)				
Whites	7 (64%)	8 (73%)	9 (82%)	
African American	4 (36%)	3 (27%)	2 (18%)	
Education^a^	3.2 ± 1.1	2.0 ± 1.3	1.4 ± 1.0	0.009
Household income^b^	2.8 ± 1.1	3.1 ± 0.7	1.9 ± 0.8	0.013
Age at first period	13.6 ± 2.3	12.6 ± 1.0	12.6 ± 1.4	0.24
Parity				
Nulliparous	5 (45%)	8 (73%)	5 (45%)	
Parous	6 (55%)	3 (27%)	6 (55%)	
Age at first birth^c^	27.5 ± 3.0	27.7 ± 4.7	23.3 ± 3.3	0.15
Breast feeding^c^	4 (67%)	2 (67%)	3 (50%)	
Family history of cancer	3 (27%)	7 (64%)	4 (36%)	

Mean ± SD are indicated. Statistical comparisons with ANOVA.

*W* non-Hispanic white American women, *AA* African American women.

^a^Education categories used: 1, less than high school; 2, high school or equivalent; 3, associate’s degree; 4, bachelor’s degree; 5, graduate degree.

^b^Income categories: 1, <$20 K/year; 2, $20–50 K/year; 3, $50–100 K/year; 4, >$100 K/year.

^c^Values for parous women.

To assess epithelial TJs, frozen tissue sections were stained for the TJ marker ZO-1. In normal differentiated epithelia, TJs form an apical barrier, visualized as continuous ‘bead-and-string’ ZO-1 signals lining the lumen. In some tissue, however, ZO-1 signals along lumens of ducts or lobules were interrupted and/or extended along basolateral cell membranes. Loss of continuity and departure from apical domains were significantly more pronounced in the high BMI group compared to the normal weight and overweight groups (Fig. 1c). While the size of adipocytes correlated with BMI, the composition of the tissue—specifically, the percentage adipose tissue area—was not related to BMI (Fig. 1d). Interestingly, epithelial polarity, based on ZO-1 distribution, positively correlated with the adipose tissue area (Fig. 1e).

Halaoui et al.^27^ have shown that lumens collapse or shrink in size during the development of breast carcinoma. To test if changes in ZO-1 localization reflect altered lumen shape, we quantified lumen sizes and the proportion of epithelial structures (acini/ducts) with a visible lumen, using H&E images of the breast tissue specimens used in Fig. 1. As shown in Supplementary Fig. 2a–c, lumen characteristics were similar across BMI groups. The continuity and radial distribution of ZO-1 signals did not correlate with lumen characteristics at the tissue level (Supplementary Fig. 2d).

We also compared the cell proliferation status in the different BMI groups using Ki67 immunostaining and found a higher proportion of epithelial structures with proliferating cells in the overweight category compared to both normal weight and obesity (Fig. 1f). The number of epithelial cell layers was used as an additional proxy to assess epithelial cell proliferation; stratified breast epithelia indeed have a higher proportion of proliferating cells^27^. For these measurements, we leveraged the Virtual Komen Tissue Bank, which enabled us to access a large independent set of images from normal breast tissue samples. Similar to Ki67 quantification, the overweight group (but not the obese group) had a slightly (~10%) higher number of cell layers compared to the normal weight group (Fig. 1g). The results indicate that breast epithelial cell polarity and, to a lesser extent, proliferation are impacted by overweight and obesity.

### Serum with metabolic hallmarks of obesity alters epithelial polarity in 3D cell culture bioassays

**Fig. 2:**
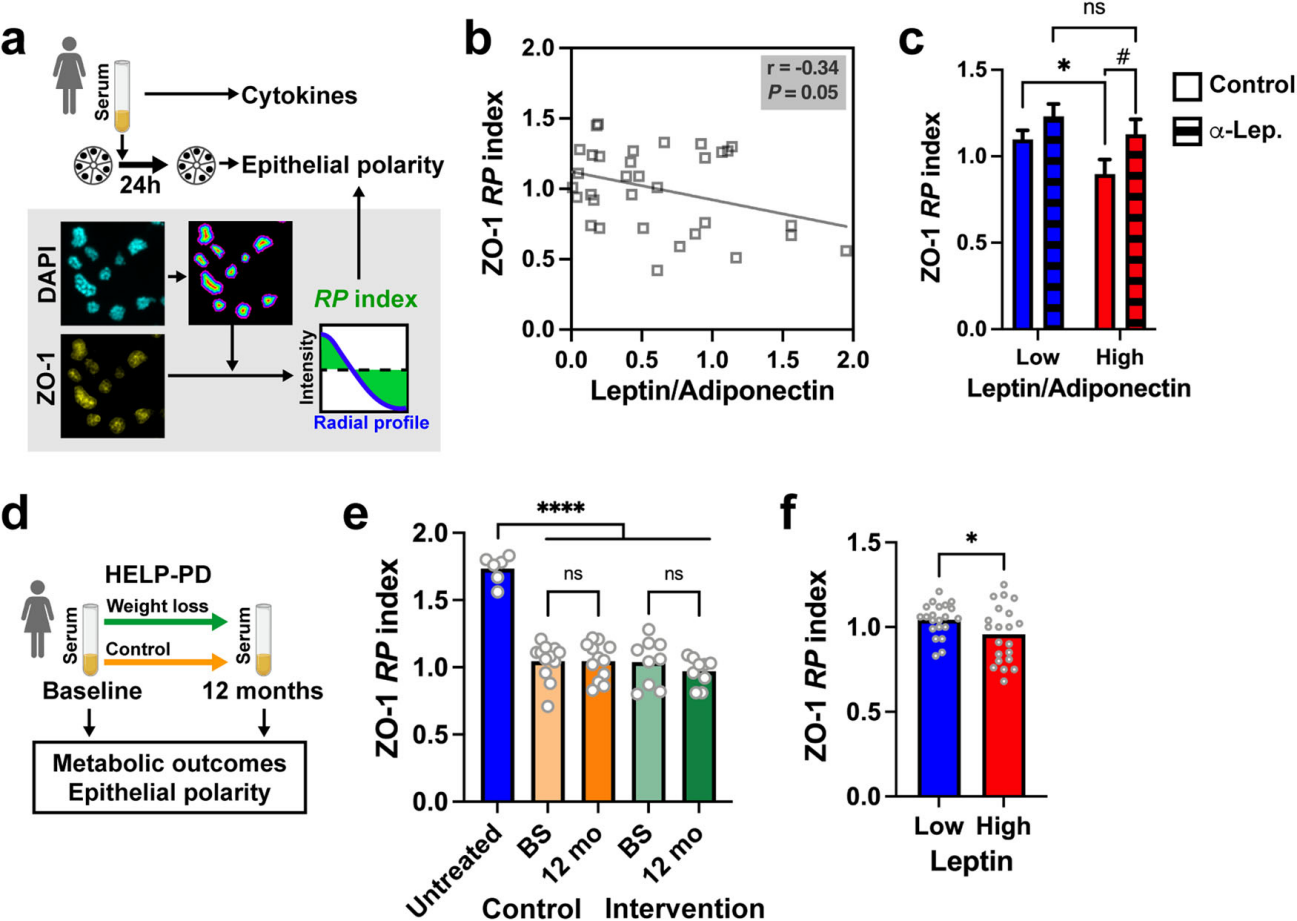
Epithelial polarity in breast acini treated with human serum. **a** Schematic of the method used to assess epithelial polarity. The radial distribution of the TJ protein ZO-1 is summarized with the *RP* index. **b** ZO-1 *RP* as a function of the leptin/adiponectin ratio in serum samples from KTB donors. *r*, Pearson’s correlation coefficient. **c** ZO-1 *RP* in acini exposed to serum with low vs high (median) leptin/adiponectin ratio. Serum was pretreated or not with leptin-neutralizing antibodies. *P < 0.05 (unpaired *t* test); ^#^*P* < 0.05 (paired *t* test); ns, not significant. Error bars represent SEM. **d** Schematic of the analysis of HELP-PD serum samples. **e** ZO-1 *RP* in acini exposed to serum from HELP-PD participants, taken at baseline (BS) or after one year of participation in the study. ****P < 0.0001; ns, not significant (ANOVA and Fischer’s LSD test). **f** ZO-1 *RP* values after classifying HELP-PD participants according to serum leptin levels. *P < 0.05 (unpaired *t* test). Symbols on the graphs correspond to the different serum samples.

Metabolic derangements characteristic of obesity may affect epithelial polarity^12^. To determine the impact of circulating factors on breast epithelial polarity, we applied serum samples from individuals with different BMI and metabolic profiles to breast acini produced in 3D cell culture (Fig. 2a). In the presence of reconstituted basement membrane (Matrigel), non-neoplastic HMT-3522 S1 breast epithelial cells develop small spheres resembling the unit of the mammary gland (the acinus). These acini are growth-arrested with apical-basal polarity^28,29^, as in the human breast. After treating the acini with serum samples from the KTB, the structures were stained for the TJ marker ZO-1 and apical polarity was assessed by measuring the radial distribution of ZO-1 fluorescent signals^30^ (Supplementary Fig. 3a, b). In polarized structures, TJ signals are localized sub-apically, very close to the center of acini which have a small lumen, thereby producing a steep radial profile (RP) in our analyses, summarized as a high *RP* index. Nonpolarized structures have a more random distribution of polarity markers that translate into flat RP profiles and lower *RP* indices. These indices correlate well with visual scores of epithelial polarity (Supplementary Fig. 3c). ZO-1 localization was not different according to KTB donor BMI (Pearson *r* = 0.094) but was inversely correlated to the leptin/adiponectin ratio measured in the serum samples (Fig. 2b). Epithelial polarity, defined by ZO-1 radial profiles, was significantly worse in acini treated with serum with high vs low leptin/adiponectin ratio, and this effect was cancelled by adding leptin-neutralizing antibodies to the serum samples (Fig. 2c).

We used the same bioassay to evaluate serum samples collected from women who participated in the HELP-PD study (the healthy living partnerships to prevent diabetes^31,32^; Fig. 2d). In this two-arm trial, adults with prediabetes were randomized to an intensive lifestyle weight-loss intervention or to a control group (enhanced usual care). The study achieved a mean 7% weight loss in women participants after one year of intervention. Although participants in the intervention arm had a greater mean reduction in BMI than participants in the control arm, weight loss outcomes were heterogeneous in both groups (Supplementary Fig. 4a). Similarly, there was an overall improvement of metabolic markers^33^, yet both positive and negative metabolic outcomes, in particular for serum leptin levels, were measured in both study arms (Supplementary Fig. 4b). Homeostatic model assessment of insulin resistance (HOMA-IR) showed significant improvements for participants in both study arms (Supplementary Fig. 4c). We measured ZO-1 radial profiles in acini treated with HELP-PD serum collected at baseline and after 12 months. There was no improvement in epithelial polarity for either the intervention or the control group (Fig. 2e). We also assessed ZO-1 radial profiles as a function of metabolic outcomes. Participants with low (below median) serum leptin had a significantly better *RP* score compared to those with high serum leptin (Fig. 2f). In contrast, *RP* indices were not different in participants with low vs. high HOMA-IR. Proliferation status of S1 acini was not affected by the incubations with the HELP-PD serum samples (Supplementary Fig. 4d, e). In summary, an in vitro assay captures metabolic properties in serum impacting epithelial polarity and effects seem to be largely driven by leptin levels.

### Loss of epithelial polarity in 3D cell culture models of obesity is reversible

**Fig. 3:**
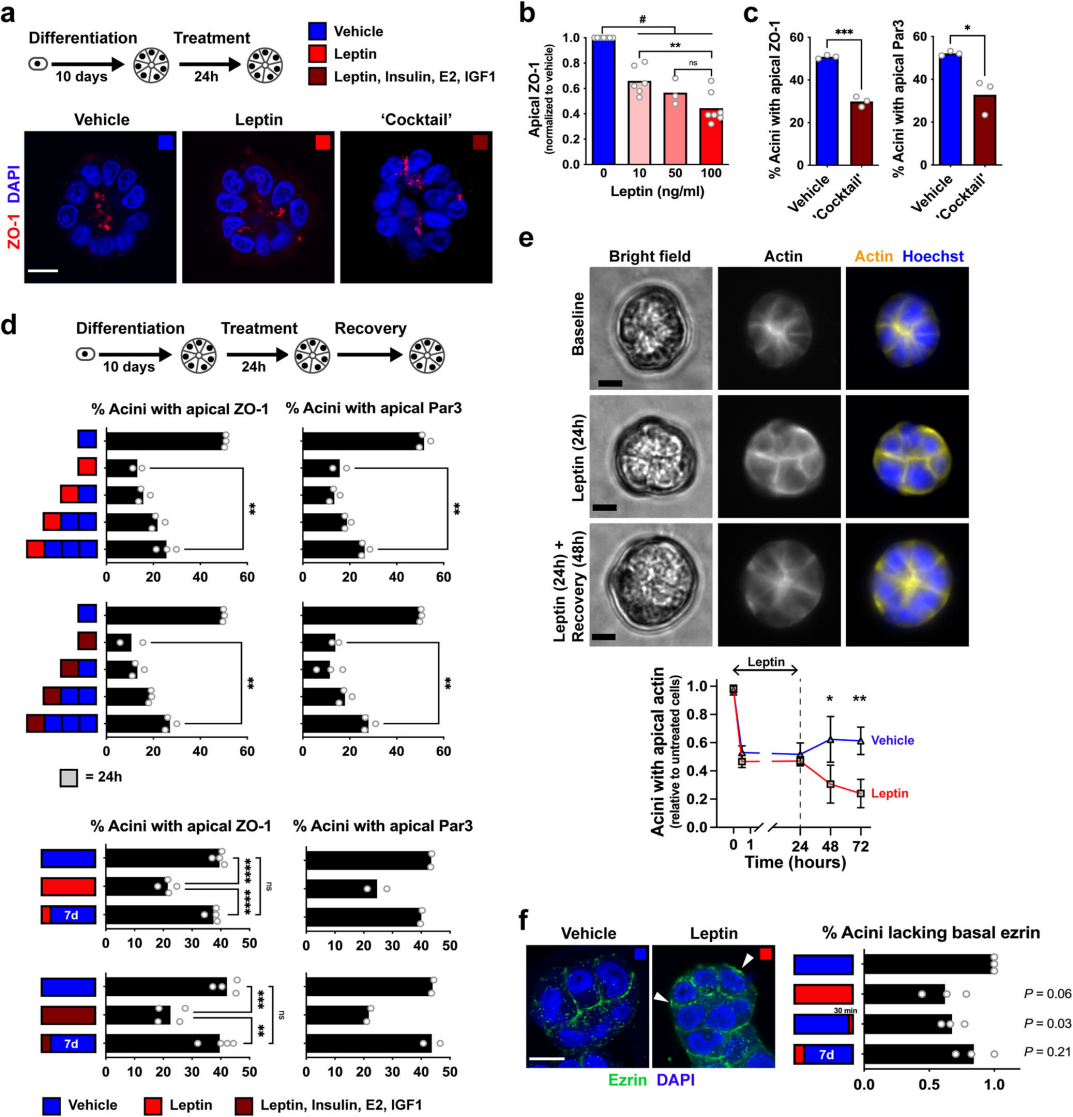
Loss of epithelial polarity in an in vitro model of adipokine imbalance is reversible. **a** Immunodetection of ZO-1 in differentiated HMT-3522 S1 acini exposed for 24 h to vehicle, leptin (100 ng/ml), or a ‘cocktail’ of adipokines, hormones, and growth factors (100 ng/ml leptin; 500 ng/ml insulin, i.e., twice the amount in H14 medium; 0.5 ng/ml β-estradiol (E2), i.e., five time the concentration in H14; and 0.1 µg/ml IGF-1). **b** Proportion of S1 acini with apical localization of ZO-1 after treatment with a range of leptin concentrations. **P < 0.005; ns, not significant (ANOVA and Tukey’s test); ^#^*P* < 0.05 (one-sample *t* test). **c** Apical localization of ZO-1 and Par3 following the combination treatment. *P < 0.05; ***P < 0.0001 (unpaired *t* test). **d** Quantification of ZO-1 and Par3 localization in acini treated with leptin or with the combination treatment, then left to recover for 24 h, 48 h, or 72 h (top), or after a longer seven days recovery period (bottom). **P < 0.01; ***P < 0.001; ****P < 0.0001 (ANOVA and Tukey’s test). **e** Quantification of cortical actin in live S1 acini. Cells were stained with Sir-Actin and the Hoechst nuclear stain, treated with leptin (100 ng/ml) or vehicle for 24 h, and imaged over a 72 h time period. Apical cortical actin is quantified in the graph. *P < 0.05; **P < 0.01 (unpaired *t* test). Representative images are shown. **f** Ezrin immunostaining of S1 acini treated with vehicle or leptin (100 ng/ml). The graph represents the proportion of structures with ezrin mislocalization at the basal domain (arrowheads) upon chronic (8 days) or acute (30 min) leptin exposure, or after leptin treatment (24 h) followed by seven days of recovery. Data are expressed relative to control. Statistics using one-sample *t* test. Scale bars: 10 µm. Symbols on the graphs represent independent experiments.

Leptin is associated with both obesity and breast cancer risk^8,34^. Our previous studies^12^ and the improvement of ZO-1 marker distribution in acini treated with serum containing leptin-neutralizing antibodies (Fig. 2c) highlight a mechanism by which leptin may promote cancer initiation in normal epithelial cells—in addition to the well-known effects of leptin on cancer cells. We confirmed that leptin levels corresponding to serum concentrations found in obesity (50–100 ng/ml) disrupt apical localization of ZO-1 in non-neoplastic acini (Fig. 3a, b and Supplementary Fig. 5a, b). This effect was dose-dependent and significantly less pronounced for cells exposed to 10 ng/ml leptin, which corresponds to the average concentration measured in serum from women in the normal weight category (Fig. 1a). Similarly, treatment of acini with a “cocktail” of adipokines, hormones, and growth factors deregulated in obesity also reduced apical localization of ZO-1. Distribution of Par3, which regulates cell–cell junctional complexes defining epithelial polarity, was also altered by this treatment (Fig. 3a, c).

Next, we asked if this effect could be reversed. Acini were exposed for 24 h to an elevated level of leptin (or to the combination treatment), leading to the disruption of apical ZO-1 and Par3 localization. The cultures were then switched back to regular medium, which lead to partial recovery of apical polarity marker distribution within 72 h, and full recovery after 7 days (Fig. 3d). As an alternative approach, we followed epithelial cell organization over a period of 3 days by labeling actin in live acini. Disruption of cortical actin organization, signifying alteration of epithelial polarity, occurred within 30 min after exposure to leptin and switching acini from leptin-containing to control medium significantly increased the proportion of structures with cortical actin rings (Fig. 3e). The actin-binding protein ezrin connects actin filaments to the plasma membrane at the apical domain of polarized epithelial cells^35^. In acini cultures, ezrin localized predominantly in the apical region, with some lateral extension of the signal, which was reminiscent of actin staining. In acini treated with leptin, ezrin signals were frequently observed at the basal membrane. This effect was partially reverted by removing leptin from the culture medium (Fig. 3f). Taken together, the data indicate that epithelial polarity of breast epithelial cells is malleable, and that alterations of apical polarity by pro-inflammatory adipokines is a rapid process which can be reverted by normalizing growth conditions.

### Restoration of epithelial polarity in a mouse model of diet-induced obesity

**Fig. 4:**
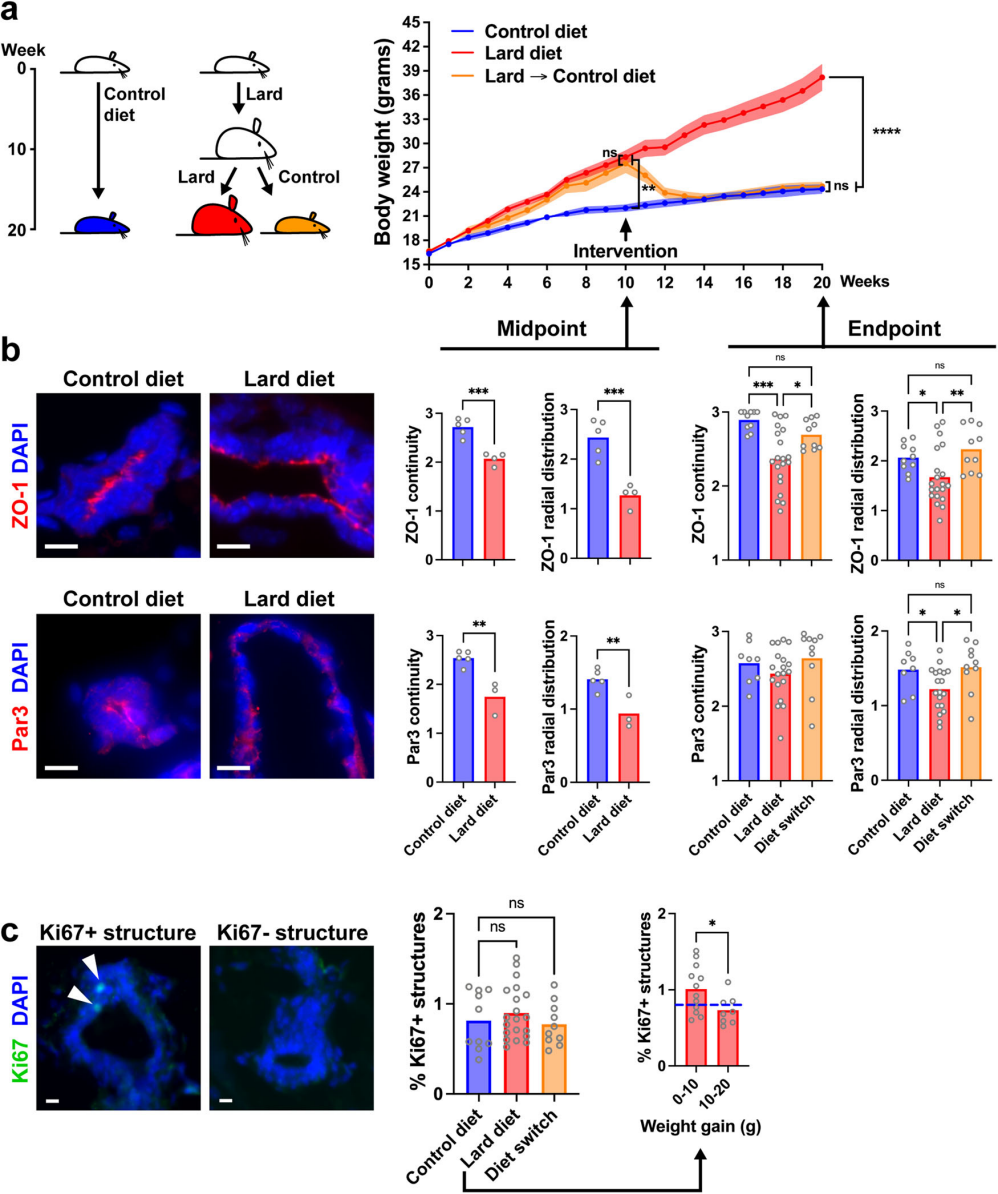
Reversion of epithelial polarity loss in mice with diet-induced obesity. **a** Body weight of C57BL/6 mice fed a control or obesity-inducing (lard) diet. The intervention group was switched from lard to control diet at the midpoint of the study, as illustrated in the schematic. ****P < 0.0001; **P = 0.001; ns, not significant (2-way ANOVA and Bonferroni’s test). **b** Detection of apical polarity markers ZO-1 and Par3 by immunofluorescence in mammary glands. Apical marker localization at the experiment midpoint and endpoint is quantified in the graphs. Representative confocal images are shown. *P < 0.05; **P < 0.01; ***P < 0.001; ns, not significant (unpaired *t* test for midpoint and ANOVA and Fischer’s LSD test for endpoint). **c** Proliferation status of mammary epithelial **c**ells assessed by Ki67 staining. Representative images of positive and negative structures are shown. Ns, not significant (ANOVA and Fischer’s LSD test). The graph on the right categorizes animals on the lard diet according to weight gain at endpoint. *P < 0.05 (unpaired *t* test). Scale bars: 10 µm. The symbols on the graphs represent individual mice.

After showing in vitro that alterations to epithelial polarity are reversible, we asked if this reversion can be achieved in vivo, using a mouse model of diet-induced obesity. Female C57BL/6 mice were fed a control diet or a lard-based high-fat diet for 10 weeks. As expected, animals on the lard diet had significantly higher body weight, body fat mass, and worse glucose tolerance compared to the control diet group (Fig. 4a and Supplementary Fig. 6a). At week 10, a portion of the animals on the lard diet were switched to the control diet, which resulted in rapid weight loss and normalization of glucose tolerance as well as whole body and mammary gland weights (Fig. 4a and Supplementary Fig. 6b). Serum leptin, which was elevated seven-fold in lard-fed mice compared to controls, returned to normal levels in the diet switch group, whereas adiponectin levels were not significantly different in the three groups (Supplementary Fig. 6b). Epithelial polarity in the mammary glands of these animals was assessed by immunostaining for apical complex proteins at the study midpoint and endpoint. The lard diet caused a reduction in continuity and luminal localization of these markers, confirming our previous observations in a different mouse strain^12,13^ (Fig. 4b and Supplementary Fig. 6c). This effect was fully reversed by the diet switch (Fig. 4b). Interestingly, there was a strong negative correlation between serum leptin and ZO-1 disruption in the lard diet group (Supplementary Fig. 6d), mirroring the dose-dependent effect of leptin on acini polarity in vitro. In the control diet group, there was an opposite correlation, with better ZO-1 polarization in the animals with higher leptin levels, which may reflect the important role of leptin in mammary gland development^36^. There was no significant correlation between mouse weight and ZO-1 distribution.

Mouse mammary gland tissue samples were stained with Ki67 to estimate epithelial cell proliferation in the different groups. There were no statistical differences between the treatment groups; the animals on the lard diet had only a slight (10%) increase in Ki67-positive cells (Fig. 4c). Nonetheless, in the lard diet group, we measured significantly more epithelial proliferation in the mammary gland from the animals which gained less weight (<10 g) compared to those that gained more weight (>10 g), which mirrors our observation in human tissue where overweight (not obesity) was associated with the highest proliferation values.

### Inhibiting ROS induction by leptin prevents loss of epithelial polarity

**Fig. 5:**
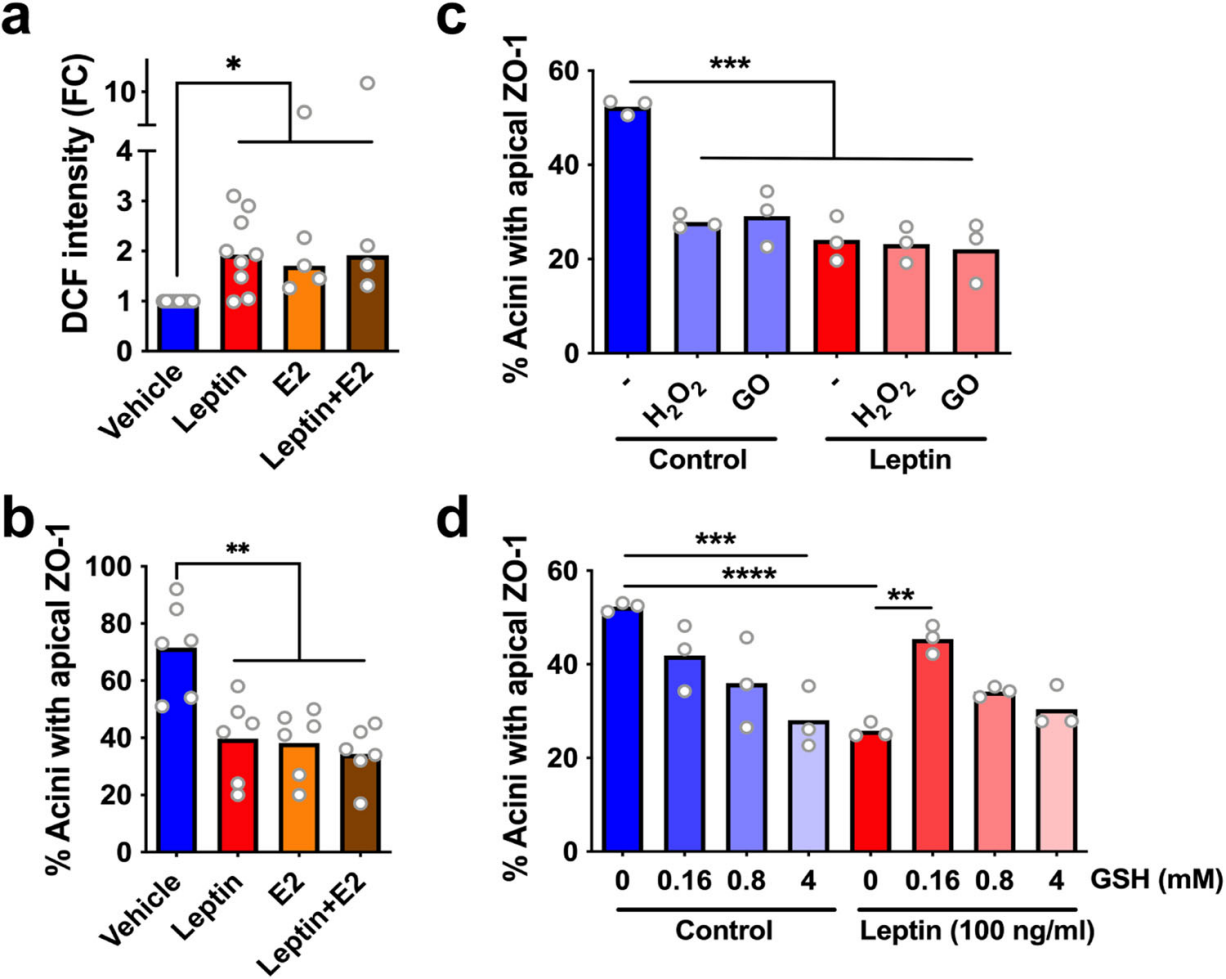
Redox control of tight junctions. **a** Quantification of reactive oxygen species with the DCF ROS sensor in acini after 30 min exposure to leptin (100 ng/ml) and/or elevated β-estradiol levels (E2; 0.5 ng/ml). *P < 0.05 (Kruskal–Wallis and Dunn’s test). **b** Proportion of S1 acini with apical localization of ZO-1 in acini treated as in **a**. **P < 0.01 (ANOVA and Tukey’s test). **c** Apical ZO-1 localization in acini after 30 min treatment with hydrogen peroxide (100 µM) or glucose oxidase (GO; 10 mU/ml), in the absence or presence of leptin. ***P < 0.001 (ANOVA and Tukey’s test). **d** Apical ZO-1 localization in acini treated with vehicle or leptin and with different amounts of glutathione (GSH). **P < 0.01; ***P < 0.001; ****P < 0.0001 (ANOVA and Tukey’s test). Symbols on the graphs correspond to independent experiments.

Leptin (as well as estrogens) rapidly generate reactive oxygen species (ROS) in various cellular contexts, including in breast epithelial cells^37–40^. We also measured significant ROS increase in breast acini acutely exposed to leptin, estradiol, or to the combination of both hormones (Fig. 5a). There was no additive effect of leptin and estrogens on ROS production. In parallel, acini cultured in medium with either leptin or increased estradiol had significantly altered ZO-1 localization (Fig. 5b). The amplitude of the effect of estradiol was similar to that of leptin and co-treatment with both factors again did not produce additive effects, suggesting convergence of the underlying mechanisms. Treating acini with oxidative stress generators (hydrogen peroxide [H_2_O_2_] or glucose oxidase [GO]) disrupted ZO-1 apical localization, to a similar extent as leptin treatments (Fig. 5c). Again, there were no additive effects on epithelial polarity in co-treatments combining leptin and H_2_O_2_ or GO.

Based on these results, we reasoned that a redox imbalance may, at least in part, explain leptin’s detrimental effect on epithelial polarity, which may be reversible by antioxidants. Indeed, glutathione and *N*-acetylcysteine counterbalanced leptin. The antioxidants reduced ROS to untreated control levels (Supplementary Fig. 7a) and partially prevented epithelial polarity loss in leptin-treated acini (Fig. 5d, Supplementary Fig. 7b). Interestingly, higher doses of antioxidants had detrimental effects on epithelial polarity. Glutathione mitigated detrimental effects on ZO-1 localization from both leptin and estrogen, suggesting a general mechanism (Supplementary Fig. 7c). While leptin treatments did not induce proliferation of acini (as reported previously^12^), estradiol caused a >2-fold increase in Ki67-positive structures, which was reverted by glutathione (Supplementary Fig. 7d).

## Discussion

Our study examined the effects of obesity on breast cancer risk and reversibility of this risk at the cellular level, by combining analyses of normal human breast tissue, a mouse model of diet-induced obesity, and 3D cell culture of breast acini. We considered two molecular markers of risk: cell proliferation and alteration of epithelial polarity, which are both necessary for breast tumor initiation. Breast tissue composition had as small but significant influence on epithelial polarity: the proportion of adipose tissue, which did not correlate with BMI nor with adipokine levels, showed a modest positive association with the integrity of cell–cell TJs. One possible explanation involves breast density. Mammographic density is an independent breast cancer risk factor^41^ and radio-dense breast tissue is characterized by low adiposity and a high proportion of stiffer extracellular matrix^42^. Stiff cellular microenvironments have been shown to compromise tissue organization^43^.

High BMI was associated with compromised epithelial polarity whereas overweight was associated with a slight increase of proliferative markers (Fig. 1). We made similar observations in mammary glands from mice fed an obesity-inducing diet (Fig. 4). As expected, leptin levels in human serum were tightly correlated with BMI whereas in mouse, leptin sharply increased in the high-fat diet group. Human serum with unfavorable adipokine levels altered epithelial polarization in a 3D cell culture bioassay of epithelial polarity (Fig. 2), mirroring the effect of leptin on cultured acini (Fig. 3). In mice fed a high-fat diet (but not in the control diet group), we measured profound alterations of apical polarity markers that correlated with serum leptin levels rather than weight gain, highlighting the importance of focusing on metabolic health^44^. Bulk tissue microarray profiling of normal breast tissue found an inverse association between the expression of adipokine receptors and of genes involved in epithelial polarity homeostasis^45^, reinforcing the notion that imbalance in adipokine signaling compromises breast epithelial polarity.

Beyond adipokines, our results indicate that other metabolic factors affect tissue architecture. Doubling estradiol levels in cultures of breast acini had the same detrimental effect as leptin on TJs. In estrogen receptor-positive breast cancer cells, estrogens disrupt of ZO-1 at the TJs, leading to nuclear relocation of the protein which promotes epithelial–mesenchymal transition^46^. Similar estrogen-induced nuclear relocation of ZO-1 and loss of TJ function was measured in endothelial cells^47^. Estrogens fluctuate naturally in the breast epithelium, and it will be interesting to assess if hyperestrogenism is associated with altered breast epithelial polarity. Diet and obesity also profoundly affect the gut and breast microbiomes, leading to elevated circulating levels of estrogens and bacterial endotoxins that negatively impact TJ functions^48–50^. Future studies should therefore address reversibility of microbiome alterations and of endotoxin-mediated changes to the mammary gland.

Apical-basal polarity is a malleable characteristic of epithelial tissue. Transient changes in epithelial polarity are an integral part of the mammary gland development, enabling branching of the ductal system^51^. Our results with 3D cultures of breast acini and diet-induced obesity in mice indicate that polarity loss due to metabolic imbalance can be reverted by normalizing the cellular microenvironment. Others found previously that oncogene-driven loss of epithelial polarity can be reverted upon de-induction of the oncogene^27^.

Re-equilibration of the redox balance disrupted by leptin, estrogens, and other pro-inflammatory cytokines may be particularly important to revert breast epithelial polarity alterations. As described previously^37–39^, we found in acute treatment settings that leptin and estradiol induce ROS in breast epithelial cells. In turn, ROS induction destabilized TJs in breast cells (Fig. 5), as previously documented in other epithelial and endothelial contexts^52^. Hence, while estrogens profoundly affect transcriptional programs important for breast epithelial homeostasis, non-transcriptional effects may also participate in pre-malignant alterations. We postulate that redox regulation of the PI3K/Akt signaling axis^53^ plays an important role in epithelial junctional complexes homeostasis since blocking this axis prevents loss of epithelial polarity by leptin^12^. Our results also indicate that low ROS levels, likely in a range conductive to signaling, are important for epithelial homeostasis.

The biphasic relationship between BMI or weight gain and proliferation in human and mouse mammary glands cannot be explained by leptin levels which were monotonically associated with BMI/weight. Also, while leptin promotes proliferation of breast cancer cells^54^, the adipokine does not induce proliferation of non-neoplastic breast epithelial cells^12,55^. In human serum analyzed in this study, some cytokines and growth factors were not monotonically associated with BMI. For example, adiponectin was lowest and IGF-1 was highest in the overweight group, which also had the highest proportion of Ki67-positive structures. Non-linear relationship between BMI and adiponectin in certain population groups has been reported^56^ and adiponectin inhibits proliferation of breast epithelial cells^57^. Fowke et al. reported peak IGF-1 levels in participants with a BMI of 22–24 kg/m^2^^58^. We showed previously that, combined with TJ disruption by leptin, exposure of breast acini to IGF-1 and insulin induces re-entry in the cell cycle^12^.

This study has several limitations. First, weight loss in the mice study was purely from a diet switch, and hence was not representative of human interventions where exercise is an important component. Moreover, since the control and obesity-inducing diets were distinct, it is possible (if not likely) that we measured a combination of obesity- and diet-related effects in these experiments. For example, the lard-based mouse diet had more cholesterol than the control diet, and dietary cholesterol is associated with breast cancer risk^11^. Also, the n-6:n-3 poly-unsaturated fatty acid (PUFA) ratio was 1.6 times higher in the lard diet compared to the control diet. High nutritional n-6:n-3 ratio is also associated with increased breast cancer risk^59^ and we showed previously that breast acini exposed to arachidonic acid (n-6 PUFA) have altered apical polarity, whereas eicosapentaenoic acid, a n-3 PUFA, has protective effects for epithelial polarity^48^. Despite its limitations, the study design enabled a clear-cut reversion of obesity/diet-induced changes, including molecular markers of breast cancer risk, which serves as proof-of-concept. Second, confounding factors may have been overlooked in our study of KTB breast tissue samples due to the small sample size. For example, although there were no clear differences in reproductive factors between BMI groups, effects linked to reproductive patterns may have been missed. There may also have been differences in socio-economic status between the BMI groups in this study, indicated by significantly lower education levels and household income for donors in the obese compared to normal weight category (Table 1).

Weight loss and lifestyle interventions leading to metabolic improvements are likely to decrease at least some molecular markers of risk at the tissue level^60^. Very few interventional studies have assessed molecular risk markers in breast tissue. The Pre-Operative Health and Body (PreHAB) window-of-opportunity study, which was conducted with breast cancer patients between diagnosis and surgery, found that exercise significantly reduces circulating leptin levels and alters gene expression in the tumors, yet breast cancer cell proliferation status was not affected by the intervention^61^. The Lifestyle, Exercise, and Nutrition (LEAN) weight loss trial with breast cancer survivors^62^ also analyzed both serum and tissue biomarkers. In contrast to PreHAB that focused on tumors, LEAN used biopsies from normal tissue. The LEAN intervention arm achieved significant weight loss and improvement in serum adipokines (similar to HELP-PD and other studies). At the tissue level, the intervention had a favorable effect on tissue inflammation (CD163) but not on proliferation (Ki67), which parallels our findings with mice (Fig. 4). Fabian et al. reported a significant effect of an aggressive weight loss intervention on proliferation (Ki67 and cyclin B1) of breast epithelial cells obtained from random peri-areolar fine-needle aspiration^63^. In this study, median weight loss in the intervention arm was 11%, which is more than in most other studies. We propose that our preclinical approach will be useful to define target improvement values, i.e., % reduction of oncogenic adipokines/cytokines, that lead to measurable changes in tissue-level risk markers. In future interventional studies, it will be important to assess tissue markers of breast cancer risk, including epithelial polarity.

## Methods

### Cell culture and treatments

Non-neoplastic HMT-3522 S1 breast epithelial cells^64^ (obtained from Dr. Mina Bissell) were propagated between passages 54 and 60 in H14 medium. 184B5 cells (obtained from ATCC) were cultured as described^65^. Routine mycoplasma tests were negative. Acinar differentiation was achieved by culturing the cells in 3D, on top of a layer of Matrigel matrix (Corning, cat# 354234), for 10 days (unless indicated otherwise), in chambered slides (MilliporeSigma, cat# PEZGS0896), as described^29^. Cells were treated with human recombinant leptin (Protein Laboratories Rehovot), leptin-neutralizing antibodies (R&D Systems, cat# AF398; 150 µg/ml), recombinant human IGF-1 (PeproTech, cat# 100-11), human recombinant insulin (MilliporeSigma, cat# 92078), β-estradiol (MilliporeSigma, cat# E2758), glutathione (MilliporeSigma), and N-acetylcysteine (MilliporeSigma), at concentrations indicated in the results section. ROS were induced by treating acini with hydrogen peroxide (H_2_O_2_) or with glucose oxidase (Sigma).

For analyses of human serum samples, a higher-throughput culture method was implemented. S1 cells were seeded in 96-well plates with glass bottom (MatTek, cat# PBK96G-1.5-5-F), at a density of 10,000 cells/well. The Matrigel coating was omitted, as done previously by Plachot et al.^66^, and acinar differentiation was achieved by adding diluted Matrigel (5% v/v) to the culture medium at the time of seeding. EGF was removed on day 3. Acini were treated on day 10 for 24 h with human serum, diluted to 1% (v/v) in H14 medium.

### Procuration of human breast tissue

Frozen needle biopsy samples of normal human breast tissues were obtained from the Susan G. Komen Tissue Bank (KTB) at the IU Simon Cancer Center (IN, USA). Data and biospecimen collection by the KTB has been approved by the Institutional Review Board of Indiana University (Protocol #1011003097). We selected 40 tissue samples donated between 2009 and 2017, from three BMI groups (normal weight [18–25]; overweight [25.5–30]; obese [>30.5]) based on the following criteria: presence of epithelium evidenced in matching H&E-stained tissue sections, availability of frozen serum samples, no hormone replacement therapy (HRT) usage, no alcohol consumption, non-smoker donor, and pre-menopausal status. We excluded HRT users, smokers, and alcohol consumers to avoid possible risk confounding factors. The BMI groups were balanced for age and race/ethnicity (Table 1). Frozen tissue sections (5 µm thick) were prepared from biopsy samples and cellularity was assessed with H&E. Thirty-three samples (83%) contained sufficient epithelium for analysis by immunofluorescence (see below). Additional analyses were done using H&E images and donor characteristics from the virtual KTB Tissue Bank (https://virtualtissuebank.iu.edu).

### Animals

The experimental protocol was approved by the Animal Care and Use Committee of the Wake Forest School of Medicine (IACUC protocol #A18-136) and all procedures were carried out in accordance with relevant guidelines and regulations. Female, 4-week-old C57BL/6 mice were purchased from Jackson. The animals were placed on a low-fat control diet (control; TD.08806) or on a lard-based obesity-inducing high-fat diet (60% kcal from fat; TD.06414), both from Envigo (Teklad diets). Nutritional characteristics of both diets are included in Supplementary Table S3. The two diets had a similar content (~2–3%) of soybean oil, but distinct n-6:n-3 fatty acid ratio and cholesterol content. Carbohydrate differences in the diets are due to corn starch composition.

At the midpoint of the study (week 10), a sub-group of animals on the high-fat diet were switched to control diet until the end of the study (week 20). Animal weights were recorded weekly. Glucose tolerance testing (GTT) was performed on fasted mice as previously described in ref. ^67^. Briefly, glucose (2 mg/kg) was administered by intraperitoneal injection and blood glucose was measured 0, 15, 30, 60, and 120 min later using a OneTouch Ultra2 (LifeScan, Inc.) and GenUltimate Test Strips.

### Analyses of serum samples

The Quantibody Human Obesity Array 3 kit (RayBiotech) was used to detect 40 adipokines in human serum samples. Analyses of existing human serum samples collected as part of the HELP-PD was approved by the Institutional Review Board at the Wake Forest School of Medicine (protocol # IRB00048164). For adiponectin, the values obtained were above the range of the standard curve and quantification was repeated using ELISA (RayBiotech). Leptin and adiponectin were quantified in mouse serum using ELISA kits from Bertin Pharma and RayBiotech, respectively.

### Immunofluorescence

Immunostaining of mammary tissue samples and of 3D cell cultures was performed as described previously^68^. Frozen tissue sections were thawed rapidly at room temperature and areas with tissue sections were delineated using a hydrophobic pen. Acini were cultured and stained in chambered slides. Samples were fixed in formalin, permeabilized with TX-100, washed in PBS-glycine, and incubated 2 h in blocking buffer [10% goat serum in immunofluorescence buffer (IF; 130 mM NaCl, 13.2 mM Na_2_HPO_4_, 3.5 mM NaH_2_PO_4_, 0.1% bovine serum albumin, 0.05% NaN_3_, 0.2% Triton X-100, and 0.05% Tween 20)]. Antibodies were diluted in blocking buffer and incubated on samples overnight at 4 °C. Samples were washed three times with IF buffer, incubated with fluorescently labeled secondary antibodies (1 h at room temperature), washed again with IF and stained with 4′,6-Diamidino-2-phenylindole dihydrochloride (DAPI; Invitrogen, 0.5 µg/ml; 10 min). Actin was stained using phalloidin-IFluor488 (AbCam). Stained sections were mounted with coverslips using ProLong Gold Antifade (Invitrogen). The following antibodies were used: claudin-1 (Abcam, Ab15098, 1 µg/ml); Ezrin (Santa Cruz Biotechnology, sc-58758, 2 µg/ml); Ki67 (Thermo Fisher Scientific, PA5-19462, 1 µg/ml); NuMA (clone B1C11, a gift from Dr. Jeffrey Nickerson, UMass, Worcester, USA); Par3 (MilliporeSigma, 07-330, 13 µg/ml); ZO-1 (Invitrogen, clone ZO1-1A12, 2.5 µg/ml). Secondary antibodies conjugated with Alexa Fluor dyes (AF488, AF568, or AF647; ThermoFisher) were used at 1:500 dilutions. For immunostaining of S1 acini in 96-well plates, fluorescently labeled primary antibodies were used: ZO-1-AlexaFluor594 (Invitrogen, clone ZO1-1A12, 1 µg/ml) and Ki67-AlexaFluor488 (CellSignalling, clone D3B5, 1:500).

### Quantification of apical polarity

In 3D cell cultures, fluorescent signals of polarity markers were scored visually for apical localization on an Olympus IX83 microscope equipped with a ×40 objective (NA = 0.95), using a binary scale. At least 100 acini were scored per condition in each experiment. Alternatively, images were recorded with a sCMOS camera (Hamamazu Orca-Flash 4.0) at ×10 magnification (NA = 0.3). Imaging of 96-well culture plates was automated using the WellNavigator function of CellSens (Olympus), with software-based autofocus on the DAPI staining. The RadialProfiler MATLAB software^30^ (https://osf.io/e37st/) was used for automated analysis of apical polarity. Briefly, RadialProfiler identifies acini based on the DAPI (or Hoechst) nuclear stain, divides the segmented acini area into concentric regions, and retrieves the intensity of the polarity marker in each of them. The intensity in each concentric layer is finally normalized to the average intensity of the entire acinus. Acini with apical polarity have high intensity values in the central regions and low intensity values at the periphery, and hence intensity profiles with a steep negative slope. A radial profile (RP) summary value is generated by integrating RP curves (Supplementary Fig. 3b).

TJ staining in human breast and mouse mammary gland tissue sections was scored visually using a 4-grade system based on the continuity at the apical ‘bead-and-string’ TJ patterns (0, <20%; 1, 20–50%; 2, 50–80%, 3, 80–100%) and the proportion of radial staining (signal radially dispersed from the apical domain; 0, >80%; 1, 50–80%; 2, 20–50%, 3 <20%). To mitigate artifacts due to tissue sectioning, only structures (ducts/lobules) with a clear lumen (based on DAPI signals) were scored. Scoring was done blind to the human donor characteristics or the mouse treatment group. Scores were validated with the analysis of a subset of tissue samples by a second scorer (Pearson’s correlation *r* = 0.72, *N* = 10). Cross-sectional area of adipocytes was measured in H&E images using a custom ImageJ (https://imagej.nih.gov/ij/) macro.

### Quantification of lumen sizes

H&E images of breast tissue sections, downloaded from the Virtual Komen Tissue Bank, were analyzed with the QuPath (https://qupath.github.io) software. Lumens were manually delineated with the polygon selection tool and measured. In addition, the proportion of acini/terminal duct sections with a discernable lumen was quantified. The absence of a detectable lumen reflects either a small or inexistent lumen, or sectioning above or below the lumen plane. We expect the proportion of the latter to be relatively constant between sections. Between 87 and 310 (median = 209) structures were analyzed for each tissue donor.

### ROS quantification

Acini were stained 5 min with 2’7’-dichlorodihydofluorescein diacetate (DCF; Invitrogen; 10 µM) and fluorescence was quantified on a plate reader, using 485/538 nm (EX/EM) filters. DCF signals were normalized to Hoechst fluorescence intensity, captured with 355/460 (EX/EM) filters. Alternatively, cells were stained for 30 min with CellROX (Invitrogen; 5 µM) and imaged with an IX83 microscope. Acini were segmented based on Hoechst staining and CellROX intensity was averaged in the corresponding regions of interest.

### Live cell imaging

Differentiated acini were stained with SiR-actin (Cytoskeleton Inc., CY-SC001, 1 µM) and Hoechst 33342 (Invitrogen, H1399, 5 µg/ml) for 1 h in a cell culture incubator. Images were collected with an IX83 wide-field microscope (Olympus), using a ×20 objective (NA = 0.45). While imaging, cells were kept at 37 °C and 5% CO_2_ with a stage-top incubator (Tokai Hit). The same regions were imaged on consecutive days by saving the frames’ *x* and *y* positions. Even illumination was achieved with a ‘Fly Eye’ fluorescence illuminator. The Cy5 filter cube (640/30 EX; 690/50 EM) was used to excite and detect SiR-actin fluorescence and the DAPI cube (350/5 EX; 460/50 EM) was used to capture Hoechst signals. Images were recorded with a Hamamazu Orca-Flash 4.0 camera.

### Statistical analysis

Statistical analyses were performed using Prism 9 (GraphPad Software Inc.). The D’Agostino & Pearson omnibus normality test was used to test for normality. Nonparametric tests were used if the data did not pass the normality test (at alpha = 0.05). The *t* test (or Mann–Whitney test) was used for comparisons between two conditions, whereas ANOVA and post hoc tests were used for datasets with more than two conditions. Statistical tests are indicated in the figure legends. All statistical tests were two-sided. A *P* value <0.05 was considered significant.

### Reporting summary

Further information on research design is available in the Nature Research Reporting Summary linked to this article.

## Supplementary information

Supplementary Information

Reporting Summary

## Data Availability

KTB donor information, available from the Virtual Komen Tissue Bank (https://virtualtissuebank.iu.edu), are provided in Supplementary Table 1. Adipokine measurements are listed in Supplementary Table 2. Imaging datasets from this study are available from the corresponding author on reasonable request.
