# Supplementary material for: Reversion of breast epithelial polarity alterations caused by obesity
Source: NPJ Breast Cancer. 2023 May 9;9:35. doi: 10.1038/s41523-023-00539-w (PMC10170133; doi:10.1038/s41523-023-00539-w)
Supplement: Supplementary file 1 — Supplementary Information [file 41523_2023_539_MOESM1_ESM.pdf]

SUPPLEMENTARY DATA

Reversion of breast epithelial polarity alterations caused by obesity

Julia Holmes, Mohamed Gaber, Mónica Z. Jenks, Adam Wilson, Tucker Loy, Cassandra Lepetit, Mara Z. Vitolins, Brittney-Shea Herbert, Katherine L. Cook, and Pierre-Alexandre Vidi

- **Supplementary Tables 1-3**
- **Supplementary Figures 1-7**

Supplementary Table 1. Characteristics of KTB donors

KTB Barcode	Age	Weight	BMI	Race	Hispanic	Education Level	Household Income	Blood Relatives Cancer
K104768	51	109	18.7	W	No	Associates Degree	$20,001 to $50,000	Yes
K106356	41	140	19	AA	No	Associates Degree	More than $100,000	No
K104993	20	111	20.3	W	No	High School Graduate or GED	$50,001 to $100,000	No
K108519	26	128	21.3	AA	No	Graduate Degree	$20,001 to $50,000	No
K105360	24	138	22.3	W	No	Graduate Degree	Less than $20,000	No
K108518	39	130	22.3	AA	No	Graduate Degree	$50,001 to $100,000	No
K106094	40	140	22.6	W	No	Graduate Degree	More than $100,000	Yes
K104563	31	141	22.8	W	No	Bachelors Degree	$20,001 to $50,000	I don't know
K105381	49	150	22.8	AA	No	Graduate Degree	More than $100,000	No
K107198	33	138	24.4	AA	No	Other	$20,001 to $50,000	I don't know
K105659	29	157	24.6	W	No	Graduate Degree	More than $100,000	Yes
K106698	21	128	25	W	No	High School Graduate or GED	$50,001 to $100,000	Yes
K105645	24	166	25.2	W	No	Bachelors Degree	$20,001 to $50,000	Yes
K108483	25	143	25.3	AA	No	Bachelors Degree	$50,001 to $100,000	Yes
K105537	23	132	25.8	AA	No	Other	$50,001 to $100,000	Yes
K104665	18	154	26.4	W	No	Less than High School	More than $100,000	No
K106635	20	136	26.6	W	No	High School Graduate or GED	$50,001 to $100,000	Yes
K104349	40	173	27.1	W	No	High School Graduate or GED	$50,001 to $100,000	No
K104146	20	156	28.5	W	No	High School Graduate or GED		Yes
K106218	41	179	28.9	W	Yes	Bachelors Degree	$20,001 to $50,000	No
K105692	44	196	29.8	AA	No	Bachelors Degree	More than $100,000	No
K104244	33	196	29.8	W	No	Graduate Degree	More than $100,000	Yes
K104729	51	180	30	W	No	High School Graduate or GED	$20,001 to $50,000	No
K104797	26	163	30.8	W	No	High School Graduate or GED	Less than $20,000	No
K106624	22	159	31	W	No	High School Graduate or GED	Less than $20,000	No
K106327	41	171	32.3	W	No	Associates Degree	$20,001 to $50,000	Yes
K104848	31	180	32.9	W	No	Vocational or technical school	$20,001 to $50,000	Yes
K105527	21	204	33.9	W	No	High School Graduate or GED	Less than $20,000	No
K106602	21	174	34	W	No	High School Graduate or GED	More than $100,000	Yes
K104949	51	239	37.4	W	No	High School Graduate or GED	$20,001 to $50,000	No
K105055	42	198	38.7	W	No	Other	$20,001 to $50,000	No
K105738	40	242	39.1	AA	No	High School Graduate or GED	$20,001 to $50,000	No
K107227	38	264	45.3	AA	No	Graduate Degree		Yes

AA, African Americans; W, Whites

Supplementary Table 1. (Cont.)

KTB Barcode	Age at First Period	Ever Been Pregnant	Currently Pregnant	Number of Pregnancies	Age at First Birth	Breast Feed (Ever)
K104768		Yes	No	2	30	Yes
K106356	19	Yes	No	2	24	No
K104993	13	No	No	0		
K108519	11	No	No	0		
K105360	12	No	No	0		
K108518	15	Yes	No	4		Yes
K106094	13	Yes	No	2	30	Yes
K104563	13	No	No	0		
K105381	13	Yes	No	4	26	Yes
K107198	14	Yes	No	1		No
K105659	11	Yes	Yes	1		No
K106698	14	No	No	0		
K105645	11	No	No	0		
K108483	12	No	No	0		
K105537	12	No	No	0		
K104665	12	No	No	0		
K106635	13	No	No	0		
K104349	14	No	No	0		
K104146	12	No	No	0		
K106218	12	Yes	No	2	26	Yes
K105692	14	Yes	No	1	33	No
K104244	12	Yes	No	2	24	Yes
K104729	13	Yes	No	2	23	No
K104797	13	Yes	No	1	22	Yes
K106624	15	No	No	0		
K106327	13	No	No	0		
K104848	11	Yes	No	1	21	No
K105527	13	No	No	0		
K106602	12	Yes	No	1	20	Yes
K104949	14	Yes	No	2	25	NO
K105055	13	No	No	0		
K105738	10	Yes	No	3	29	Yes
K107227	11	No	No	0		

Supplementary Table 2. Cytokine/adipokine/growth factor levels in serum from KTB tissue donors

KTB Barcode	Adiponectin (ug/ml)	Adipsin (ng/ml)	AgRP (ng/ml)	ANGPTL4 (ng/ml)	BDNF (ng/ml)	Chemerin (ng/ml)	CRP (ng/ml)	GH (ng/ml)	IFNg (pg/ml)	IGFBP-1 (ng/ml)
K104146	50.1	8.0	0.29	17.5	18.2	6.9	7.0	2.37	5.2	42.3
K104244	34.1	7.2	0.23	14.6	20.4	10.3	13.8	0.13	18.0	11.5
K104349	20.9	7.5	0.34	424.7	39.0	8.0	8.7	1.20	16.2	2.1
K104563	53.2	8.0	0.41	32.1	17.0	9.3	16.4	0.09	19.4	11.2
K104665	25.4	8.5	0.73	19.9	9.2	12.3	11.7	0.45	20.0	2.3
K104729	48.4	6.9	0.29	5.5	16.7	11.0	9.7	0.53	12.8	12.9
K104768	113.5	7.7	0.13	6.5	22.1	5.2	15.8	2.69	12.0	14.0
K104797	31.6	8.0	0.34	1.8	24.6	12.4	17.5	2.81	22.7	13.1
K104848	12.8	7.6	0.19	7.2	13.5	7.2	16.7	0.18	14.5	3.4
K104949	39.7	8.6	0.27	11.0	9.1	8.9	16.7	0.35	13.8	5.3
K104993	131.0	7.9	0.09	14.7	12.4	9.2	14.7	0.01	2.7	25.8
K105055	76.2	8.1	0.19	247.3	12.8	8.0	14.6	0.01	19.0	2.1
K105360	84.6	6.2	0.02	1.2	7.7	4.8	15.2	0.26	17.9	23.8
K105381	51.2	7.4	0.08	7.9	12.1	5.8	10.7	2.77	8.6	11.4
K105527	35.3	9.2	0.24	4.1	26.6	9.3	14.6	3.22	30.7	11.9
K105537	17.6	9.6	0.30	348.4	21.6	15.1	20.9	0.07	41.9	2.2
K105645	15.9	8.0	0.25	16.5	28.5	9.3	15.4	0.11	13.5	35.1
K105659	41.0	10.0	0.21	7.6	12.3	9.9	17.2	0.89	16.5	53.4
K105692	89.1	10.5	1.01	6.1	27.4	11.2	17.4	0.47	25.9	10.1
K105738	83.0	8.4	0.18	11.0	23.3	9.5	15.9	2.17	16.4	3.7
K106094	85.8	7.7	0.28	45.2	10.9	5.3	15.0	0.44	14.9	28.9
K106218	41.4	9.1	0.06	56.5	12.3	8.5	15.2	0.09	20.1	1.6
K106327	53.7	8.9	0.51	70.4	50.0	5.9	18.9	0.16	19.5	39.4
K106356	65.1	9.0	0.44	42.6	24.4	7.9	13.7	0.24	14.2	31.4
K106602	53.0	9.9	0.17	24.9	19.7	8.9	13.1	0.04	72.5	15.9
K106624	169.3	8.8	0.09	28.6	16.9	7.4	15.4	0.11	0.4	23.2
K106635	48.3	9.4	0.42	162.1	35.8	11.3	21.6	1.18	14.0	36.2
K106698	64.8	10.5	0.45	67.1	29.7	10.3	16.8	3.32	16.4	14.0
K107198	22.7	9.1	0.37	19.3	29.4	8.9	19.5	0.13	16.5	15.8
K107227	49.4	9.6	0.21	15.4	58.2	15.6	21.2	0.03	25.9	5.6
K108483	31.8	11.4	0.03	16.5	34.9	8.0	12.7	1.66	12.6	1.5
K108518	69.3	8.2	0.08	14.1	42.8	10.0	11.0	2.39	13.5	12.8
K108519	115.7	11.4	0.88	132.8	12.3	17.9	31.6	0.82	56.9	16.8
LOD	*N.A.*	*0.06*	*0.12*	*0.27*	*0.06*	*0.50*	*0.01*	*0.01*	*9.62*	*0.02*
HS	*N.A.*	*40*	*20.00*	*200*	*80*	*200*	*40*	*40.00*	*20000*	*40*

LOD, limit of detection; HS, highest standard

AgRP, Agouti-related protein; ANGPTL4, Angiopoietin-related protein 4; BDNF, Brain-derived neurotrophic factor; CRP, C-reactive protein; GH, Growth hormone; IFNg, Interferon gamma; IGFBP-1, Insulin-like growth factor-binding protein 1

Supplementary Table 2. (cont.)

KTB Barcode	IGFBP-2 (ng/ml)	IGF-1 (ng/ml)	IL-10 (pg/ml)	IL-12p40 (pg/ml)	IL-12p70 (pg/ml)	IL-1b (pg/ml)	IL-1ra (pg/ml)	IL-6 (pg/ml)	IL-8 (pg/ml)	Insulin (ng/ml)
K104146	18.9	314.4	3.31	84.9	0.7	4.9	524.6	180.7	3.4	2.0
K104244	18.3	332.8	1.99	123.1	5.9	21.6	741.1	156.0	2.0	3.7
K104349	15.7	351.4	4.41	255.9	7.2	14.6	1034.8	174.2	9.9	4.9
K104563	16.5	316.6	2.74	95.8	6.5	11.4	665.9	214.8	7.2	4.5
K104665	17.6	388.6	2.87	133.3	16.9	20.2	542.3	166.2	10.7	4.1
K104729	17.4	255.5	9.29	122.0	1.7	19.1	707.0	159.0	15.1	4.5
K104768	18.1	255.5	3.64	118.7	20.0	10.7	369.6	219.3	1.0	3.0
K104797	15.8	319.6	1.35	176.1	23.7	33.2	141.7	149.6	7.5	3.0
K104848	14.9	297.9	2.77	74.5	5.8	14.2	272.2	210.4	7.9	14.8
K104949	18.7	322.1	2.80	196.4	3.4	4.9	808.0	187.0	3.6	2.3
K104993	12.6	312.8	3.50	134.3	2.8	16.4	103.3	170.3	6.9	2.3
K105055	18.1	338.8	4.34	253.6	1.2	0.0	1272.0	215.1	3.2	3.8
K105360	13.8	178.2	2.39	4.9	0.0	0.0	45.6	211.4	0.8	0.2
K105381	14.9	215.7	0.95	66.7	0.0	0.0	37.3	126.4	0.0	0.0
K105527	13.3	296.9	5.94	173.3	10.6	1.8	582.8	225.3	11.5	3.3
K105537	17.0	420.8	6.56	55.4	7.2	6.9	389.8	215.3	16.4	2.4
K105645	15.6	273.3	7.05	232.1	5.2	5.5	123.3	194.8	6.4	4.1
K105659	27.6	434.4	6.86	222.2	5.6	17.3	318.3	177.5	4.3	1.1
K105692	18.7	317.3	6.02	252.5	17.6	10.7	219.0	230.8	11.7	2.0
K105738	17.3	469.6	2.89	97.0	4.1	1.3	491.1	222.7	9.0	3.9
K106094	15.2	264.2	3.53	131.3	18.2	0.0	804.3	230.0	11.1	4.1
K106218	16.6	348.0	4.75	102.2	3.6	0.0	889.3	169.4	8.2	7.8
K106327	15.7	308.0	5.36	217.2	7.6	2.1	370.5	158.6	8.6	4.3
K106356	19.0	298.8	2.52	164.0	1.5	5.3	712.5	201.7	3.1	5.9
K106602	18.6	407.1	6.44	252.6	42.4	0.9	1073.7	193.0	31.8	8.9
K106624	21.8	344.2	0.00	113.8	2.2	0.0	367.8	199.4	4.8	2.9
K106635	21.8	366.4	3.91	242.3	0.0	0.0	218.6	204.1	7.4	4.6
K106698	14.7	363.8	5.71	184.2	0.0	8.1	256.2	139.0	4.0	3.3
K107198	17.5	428.5	4.24	162.9	78.0	13.7	2178.3	181.7	22.1	4.9
K107227	23.7	450.4	6.34	47.1	2.7	7.2	671.3	210.8	4.9	5.4
K108483	28.8	462.9	3.89	323.8	4.8	1.6	672.0	244.5	9.9	9.6
K108518	14.9	262.0	2.50	71.7	1.3	10.3	189.0	145.6	3.6	3.0
K108519	25.6	622.0	8.90	189.7	11.6	36.9	498.2	207.0	36.2	9.8
LOD	*0.03*	*5.02*	*1.52*	*22.90*	*4.49*	*9.38*	*125.23*	*19.18*	*5.54*	*2.57*
HS	*40*	*400*	*4000.00*	*20000*	*4000*	*4000*	*40000*	*4000*	*800*	*400*

LOD, limit of detection; HS, highest standard
IGFBP-2, Insulin-like growth factor-binding protein 1; IGF-1, Insulin-like growth factor I; IL-, Interleukin-;

Supplementary Table 2. (cont.)

KTB Barcode	Leptin (ng/ml)	Lipocalin-2 (ng/ml)	MSP (ng/ml)	OPG (ng/ml)	PAI-1 (ng/ml)	PDGF-BB (ng/ml)	Pepsinogen I (ng/ml)	Pepsinogen II (ng/ml)	Procalcitonin (pg/ml)	Prolactin (pg/ml)
K104146	30.7	1.2	6.3	3.8	33.1	5.4	2.3	2.7	0.0	231.3
K104244	32.3	1.2	1.9	2.8	18.5	4.6	2.6	2.1	0.0	139.2
K104349	32.5	1.4	11.1	21.7	29.9	5.1	2.1	1.8	0.0	186.9
K104563	10.7	1.2	2.3	4.8	19.3	4.2	3.2	1.8	0.0	30.4
K104665	13.0	1.2	1.7	6.7	23.0	4.2	2.2	0.7	850.0	240.0
K104729	21.0	1.3	3.6	2.6	22.5	4.1	2.5	0.9	0.0	285.6
K104768	1.5	1.2	7.8	6.2	30.5	6.1	3.6	6.4	72.7	178.9
K104797	24.3	1.2	8.0	1.2	15.9	4.3	3.0	2.3	716.4	529.0
K104848	24.9	1.2	6.9	3.8	20.0	6.2	2.7	3.6	0.0	85.0
K104949	24.3	1.1	10.4	2.9	20.2	5.7	2.5	4.6	0.0	588.7
K104993	6.1	1.3	4.2	3.4	20.4	6.0	2.9	2.7	340.6	82.5
K105055	36.8	1.3	8.5	14.0	15.3	4.5	2.2	2.3	0.0	170.3
K105360	12.8	0.7	5.0	1.6	21.4	5.5	3.0	4.3	0.0	232.5
K105381	7.3	0.9	1.4	1.6	9.5	3.1	1.9	1.3	0.0	156.5
K105527	39.0	1.4	2.9	1.9	24.0	5.6	2.3	1.3	189.5	399.8
K105537	27.5	1.3	1.8	33.2	18.4	4.2	2.0	2.3	1848.3	818.2
K105645	18.6	1.3	9.8	5.3	20.7	6.6	2.9	2.7	0.0	201.2
K105659	7.2	1.4	3.1	2.7	17.4	4.3	2.6	1.6	0.0	1009.6
K105692	39.2	1.4	9.2	2.8	19.5	5.3	1.9	1.3	802.0	292.8
K105738	54.4	1.6	5.2	3.2	16.2	5.2	2.5	2.5	0.0	111.0
K106094	16.8	1.4	0.7	10.0	23.6	4.4	3.2	4.1	201.0	322.9
K106218	38.1	1.3	1.1	6.9	16.6	4.8	3.6	5.1	0.0	343.4
K106327	20.7	1.1	1.8	9.4	23.0	6.4	2.6	2.1	1845.7	333.8
K106356	2.3	1.1	1.3	5.7	19.8	4.0	2.4	3.0	0.0	229.0
K106602	46.7	1.3	8.4	3.0	24.9	5.2	2.6	1.3	0.0	97.6
K106624	9.9	1.2	2.2	4.6	14.6	4.6	2.2	2.8	0.0	241.2
K106635	20.1	1.3	27.0	11.2	27.5	6.6	3.4	4.3	0.0	249.7
K106698	12.2	1.2	1.8	4.0	19.2	4.1	2.6	3.0	0.0	749.6
K107198	26.0	1.3	2.3	4.0	19.4	5.8	3.9	8.4	0.0	683.4
K107227	52.7	1.7	1.1	2.2	23.0	5.1	2.2	2.4	0.0	379.6
K108483	30.4	1.7	3.3	8.9	30.0	6.7	2.3	3.0	0.0	466.1
K108518	10.8	1.1	0.9	2.6	17.2	3.9	2.1	2.2	0.0	64.9
K108519	16.6	1.9	2.4	7.3	29.0	6.2	4.4	4.2	689.1	790.3
LOD	*0.07*	*0.00*	*0.01*	*0.14*	*0.17*	*0.01*	*0.01*	*0.03*	*262.59*	*8.78*
HS	*80*	*4*	*200*	*40*	*200*	*4*	*40*	*80*	*200000*	*8000*

LOD, limit of detection; HS, highest standard
MSP, Macrophage stimulatory protein; OPG, Osteoclastogenesis inhibitory factor; PAI-1, Plasminogen activator inhibitor 1; PDGF-BB, Platelet-derived growth factor subunit B

Supplementary Table 2. (cont.)

KTB Barcode	RANTES (ng/ml)	RBP4 (ng/ml)	Resistin (ng/ml)	SAA (ng/ml)	TGFb1 (pg/ml)	TSP-1 (ng/ml)	TNF RI (ng/ml)	TNF RII (ng/ml)	TNFa (pg/ml)	VEGF (pg/ml)
K104146	1.4	13.6	7.1	52.7	0.0	5618.3	6.5	2.6	0.0	260.3
K104244	1.3	10.8	4.8	55.8	397.1	4997.5	3.9	2.2	926.9	443.1
K104349	1.5	13.6	17.7	60.7	0.0	5619.4	6.3	2.1	163.3	437.5
K104563	1.3	11.7	5.9	66.1	1132.0	5170.1	6.0	2.1	603.3	398.4
K104665	1.5	13.3	8.1	65.0	4088.8	6307.6	7.1	2.9	981.4	499.0
K104729	1.2	12.6	4.4	58.0	0.0	5377.8	6.2	2.9	817.5	394.2
K104768	1.1	8.6	2.4	47.6	0.0	4266.2	3.8	1.9	0.0	360.3
K104797	1.1	9.0	5.5	58.8	0.0	4414.2	3.4	2.3	1759.5	623.0
K104848	1.3	9.2	9.4	55.8	252.3	5266.7	3.1	2.5	991.8	679.2
K104949	1.1	9.9	5.6	54.1	0.0	5537.8	7.0	3.6	170.4	1102.4
K104993	1.2	11.1	9.2	49.0	0.0	4943.7	3.6	2.0	91.0	962.8
K105055	1.2	9.1	6.0	29.5	0.0	5170.3	6.4	2.4	0.0	288.3
K105360	1.3	10.1	1.3	52.1	0.0	4373.2	0.9	0.6	257.4	374.7
K105381	0.9	7.0	6.0	30.6	0.0	3548.1	3.2	1.4	0.0	130.2
K105527	1.3	10.1	36.1	66.5	2033.0	5243.7	6.4	2.6	126.9	222.7
K105537	1.1	8.0	7.0	43.1	155.0	4815.3	3.3	1.6	735.2	534.2
K105645	1.2	12.2	2.6	55.4	467.9	5076.6	4.3	3.2	39.7	672.9
K105659	1.1	11.3	7.0	50.0	0.0	5155.4	6.9	4.2	19.9	100.0
K105692	1.2	13.9	10.4	36.8	2403.1	4928.8	5.9	3.0	1125.4	1082.5
K105738	1.2	12.5	7.3	33.7	0.0	5527.6	5.5	1.8	252.8	314.9
K106094	1.4	10.5	5.1	74.0	1365.0	5066.4	4.0	2.4	382.5	329.1
K106218	1.1	8.6	4.4	125.8	663.8	4682.1	4.3	1.7	0.0	400.6
K106327	1.3	10.0	7.5	65.1	3871.0	5076.0	5.4	2.8	1379.9	485.9
K106356	1.2	9.4	8.5	59.2	486.1	5129.4	5.7	2.4	0.0	28.8
K106602	1.3	10.8	5.3	54.0	23894.9	5667.2	4.6	3.0	717.1	449.2
K106624	1.2	9.4	2.4	68.7	249.1	4770.8	3.2	1.8	0.0	276.1
K106635	1.3	11.9	4.7	57.6	169.0	5432.4	7.6	3.7	439.2	624.1
K106698	1.1	9.6	7.8	66.3	0.0	4618.1	6.5	2.6	0.0	337.9
K107198	1.2	12.7	4.0	65.8	4035.2	5426.9	4.4	3.9	2763.5	276.7
K107227	1.2	10.5	10.2	62.8	0.0	5520.7	7.7	2.8	224.4	245.0
K108483	1.7	14.6	9.2	67.9	2315.4	6928.7	4.7	2.2	1374.3	1335.5
K108518	1.1	9.8	2.3	54.0	1132.9	4096.4	4.3	1.8	120.6	686.6
K108519	1.3	17.6	7.8	62.1	8959.7	6810.8	8.9	4.8	5089.1	1570.0
LOD	*0.00*	*0.01*	*0.03*	*0.10*	*1711.93*	*20.32*	*0.01*	*0.01*	*614.74*	*52.06*
HS	*4*	*20*	*80*	*200*	*200000*	*400*	*200*	*4*	*4000*	*20000*

LOD, limit of detection; HS, highest standard
RBP4, Retinol-binding protein 4; SAA, Serum amyloid A-1 protein; TGFb1, Transforming growth factor beta-1; TSP-1, Thrombospondin-1; TNF RI/II, Tumor necrosis factor receptor superfamily member 1A/1B; TNFa, Tumor necrosis factor alpha; VEGF, Vascular endothelial growth factor A

Supplementary Table 3. Nutritional characteristics of the experimental diets

	Control diet*	Lard diet**
Protein (% kcal)	20.5%	18.3%
Carbohydrates (% kcal)	69.1%	21.4%
Fat (% kcal)	10.5%	60.3%
Kcal/gram	3.6	5.1
Saturated fat	26.5%	36.1%
Monounsaturated fat	32.7%	40.6%
Polyunsaturated fat	40.8%	23.4%
n-6:n-3 fatty acid ratio	7.9	13
Sucrose (% by weight)	11%	12%
Cholesterol (mg/kg)	61	347.5
Sodium (g/kg)	1.0	1.4

Diet sources: * TD.08806 and **TD.06414 from Envigo.

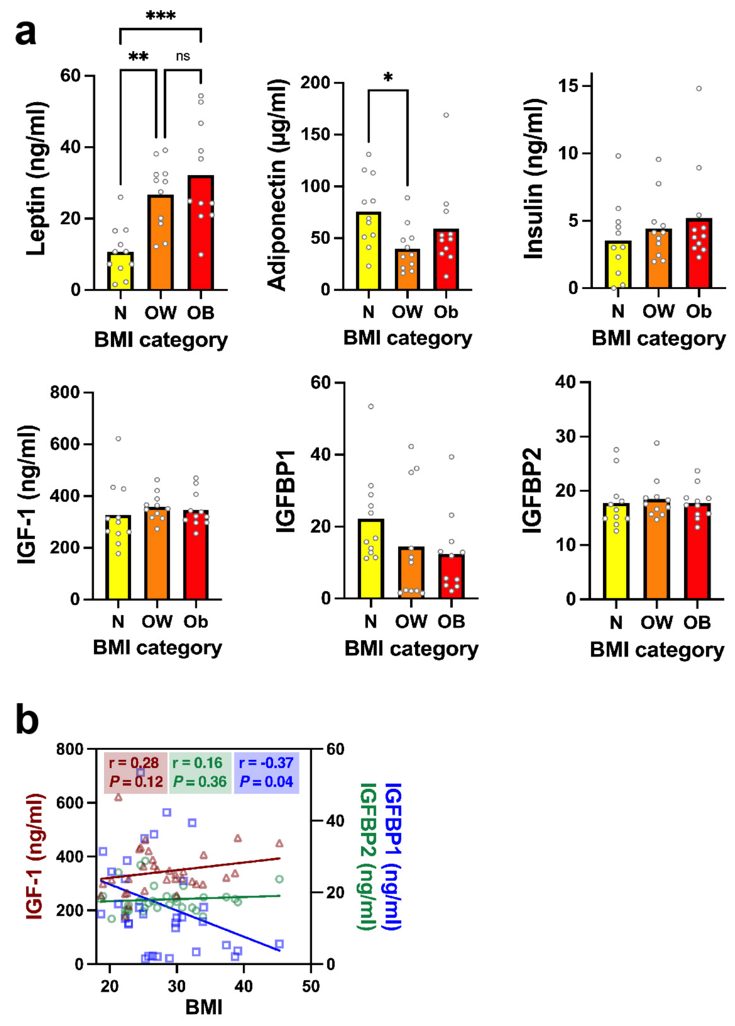

Supplementary Figure 1. Serum factor levels from KTB donors with different BMI. a Average levels of leptin, adiponectin, insulin, IGF-1, and IGF binding proteins 1 and -2 in the three BMI categories (N, normal weight; OW, overweight; OB, obese). *, $P < 0.05$; **, $P < 0.01$; ***, $P < 0.001$ (ANOVA and Tukey's test). **b** Levels of IGF1, IGFBP1, and IGFBP2 as a function of BMI. Spearman correlation coefficients (r) are indicated with corresponding *P*-values.

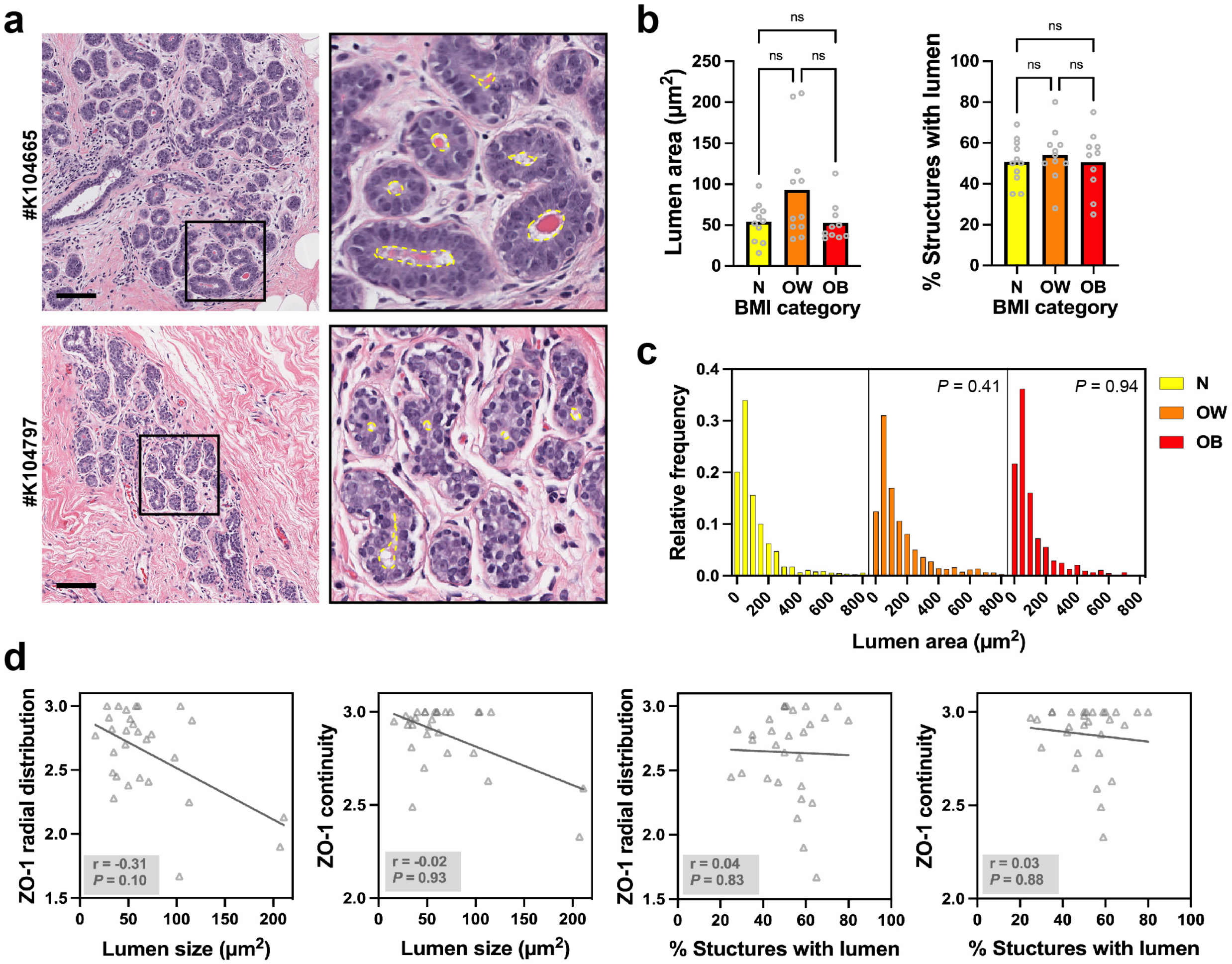

Supplementary Figure 2. Lumen sizes. a Representative images of two H&E-stained sections of breast tissue from KTB donors, illustrating different lumen sizes (yellow dashed lines in enlarged images). Scale bars, 100 μm. **b** Median lumen size (left) and proportion of structures with a visible lumen (right) as a function of BMI categories (N, normal weight; OW, overweight; OB, obese). Ns, not significant (Kruskal-Wallis and Dunn's test). Each symbol on the graphs represents a tissue donor. Ns, not significant. **c** Frequency distribution of lumen sizes in the different BMI categories. All measurements from all donors in each BMI category were combined. *P* values from Mann Whitney tests (vs. N). **d** Radial distribution and continuity of ZO-1 immunostaining signals as a function of median lumen size or of the proportion of structures with a visible lumen. Spearman correlation coefficients (r) and corresponding *P*-values are indicated.

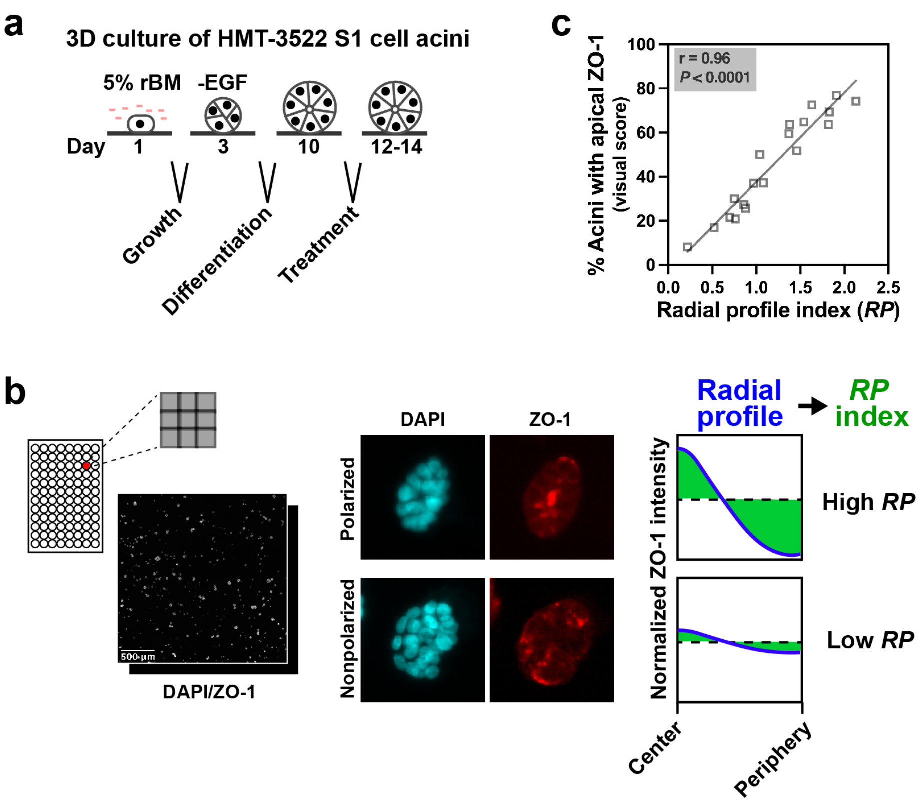

Supplementary Figure 3. Automated quantification of epithelial polarity in breast acini. a Breast epithelial cells (HMT-3522 S1) are cultured directly in 3D on glass bottom 96-well plates, without a layer of reconstituted basement membrane (Matrigel). Rather, diluted Matrigel is added to the culture medium on day 1. EGF is removed on day 3 to promote acinar differentiation and avoid cell spreading as monolayers. **b** Acini stained with DAPI (cell nuclei, used for segmentation) and for the TJ marker ZO-1 are imaged with an automated microscope (9 fields per well, 10x magnification). Representative images of non-polarized and polarized acini are shown. The cartoon illustrates radial distributions of ZO-1 staining intensity, from the center to the periphery of the structures. Radial intensity profiles are used to compute *RP* index metrics. **c** Correlation between *RP* values and visual scores of apical ZO-1 distribution. Each symbol represents a treatment condition, with 100 - 380 acini/condition. r, Pearson's correlation coefficient.

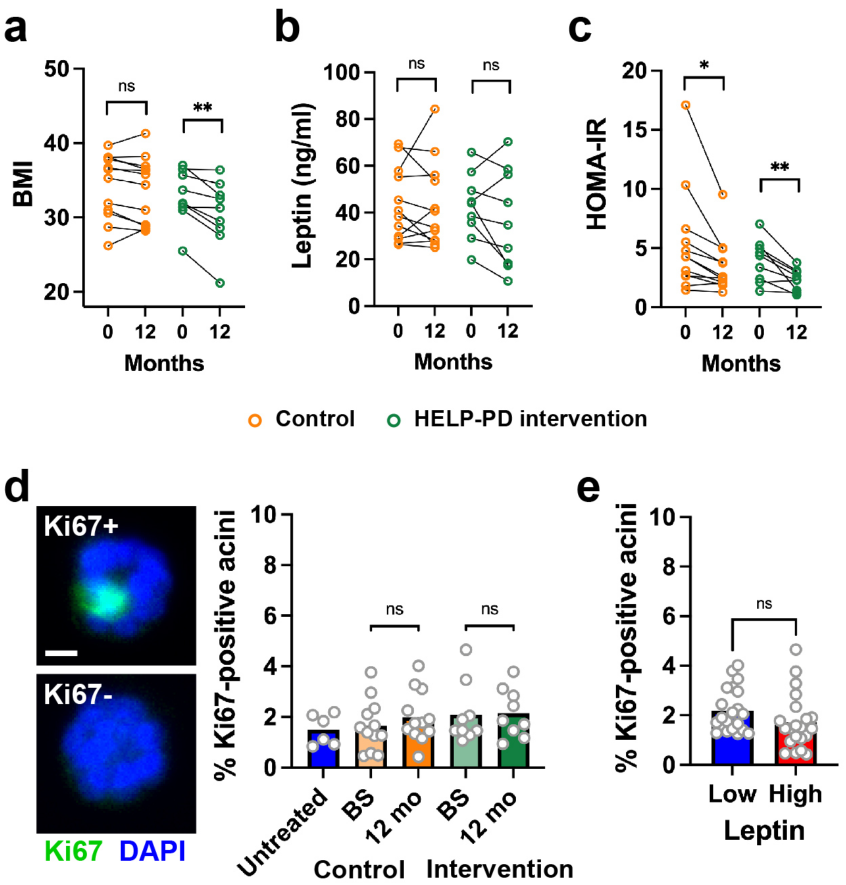

Supplementary Figure 4. Outcomes of the HELP-PD study. BMI (**a**), serum leptin (**b**), and homeostatic model assessment of insulin resistance (HOMA-IR; **c**) from female participants to the HELP-PD study, by study arm (control and intervention), at baseline (0 month) and at the 1-year time point. *, $P < 0.05$; **, $P < 0.01$; ns, not significant (paired t-test). **d** Proliferation status of S1 acini treated for 24h with serum from HELP-PD participants. BS, baseline. Ns, not significant (paired t-test). Representative images of positive and negative acini are shown. Scale bar, 10 µm. **e** Proportion of Ki67-positive acini after classifying HELP-PD participants according to serum leptin levels. ns, not significant (Mann-Whitney).

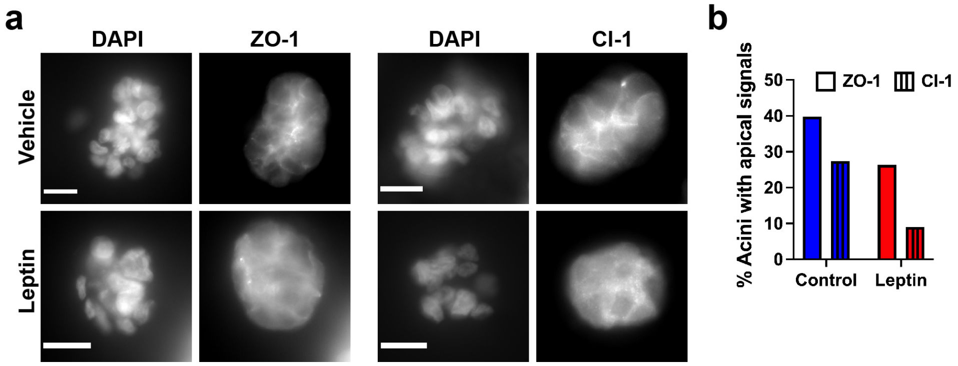

Supplementary Figure 5. Loss of epithelial polarity in 184B5 cell acini exposed to leptin. 3D cultures were treated for 24h with vehicle or leptin (100 ng/ml). **a** representative images of cell nuclei (DAPI) and of immunostaining for ZO-1 and claudin-1 (Cl-1). Scale bars, 20 µm. **b** Proportion of S1 acini with apical localization of ZO-1 and Cl-1.

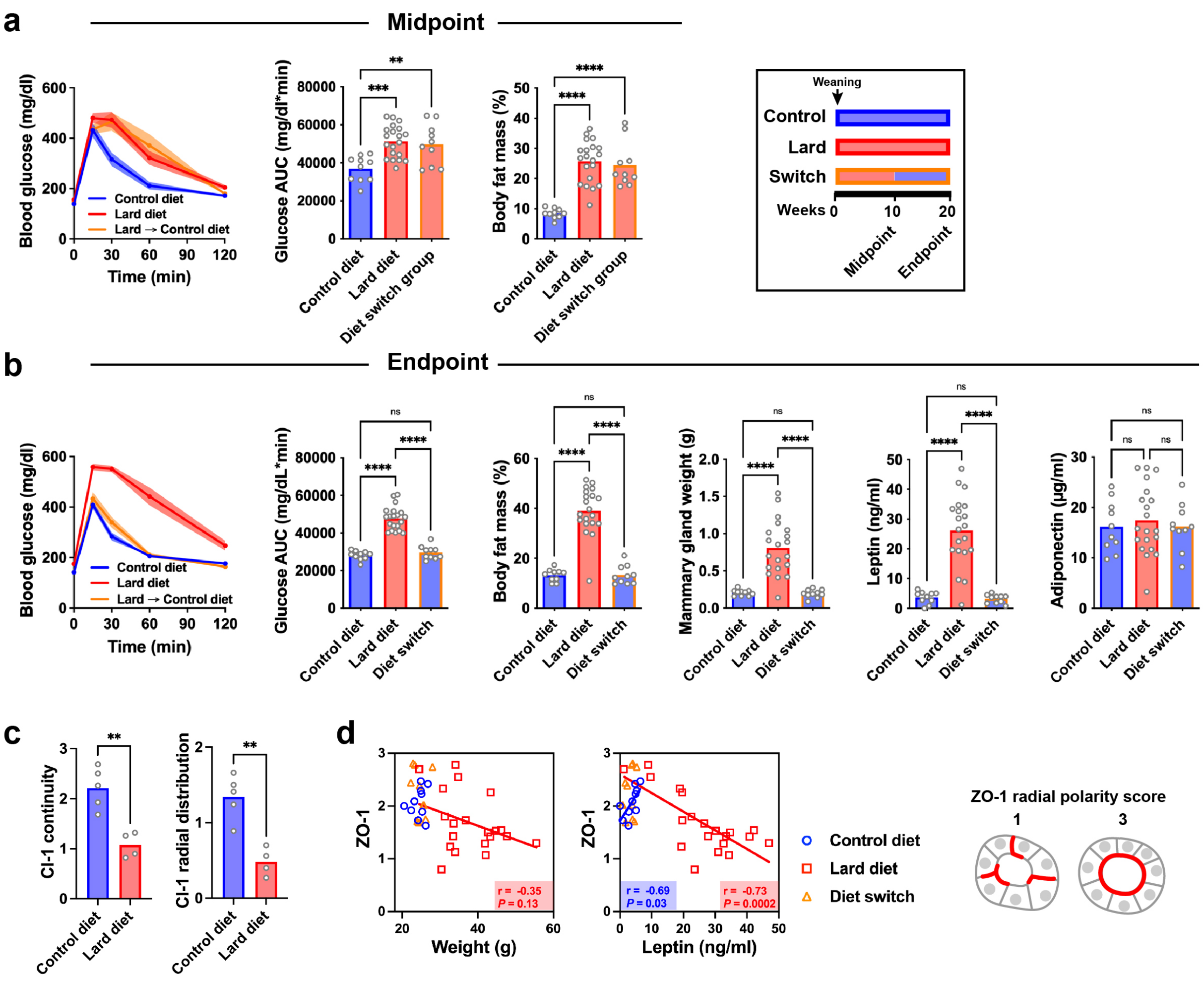

Supplementary Figure 6. Body and metabolic characteristics of C57BL/6 mice fed control and lard diets. a Measurements of glucose tolerance and body fat mass at the midpoint, before diet switch in the intervention group. **b** Measurements of glucose tolerance, body fat, mammary gland weights and serum adipokine levels at the endpoint, 10 weeks after switching mice from lard to control diet in the intervention group. AUC, area under curve. **c** Quantification of apical localization of claudin-1 at the study midpoint. **d** Radial distribution of ZO-1 in the mouse mammary glands at the endpoint as a function of animal weight or serum leptin concentration. r, Pearson's correlation coefficient. **, $P < 0.01$; ***, $P < 0.001$; ****, $P < 0.0001$; ns, not significant (ANOVA and Tukey's test [**a** and **b**] and unpaired t-test [**c**]).

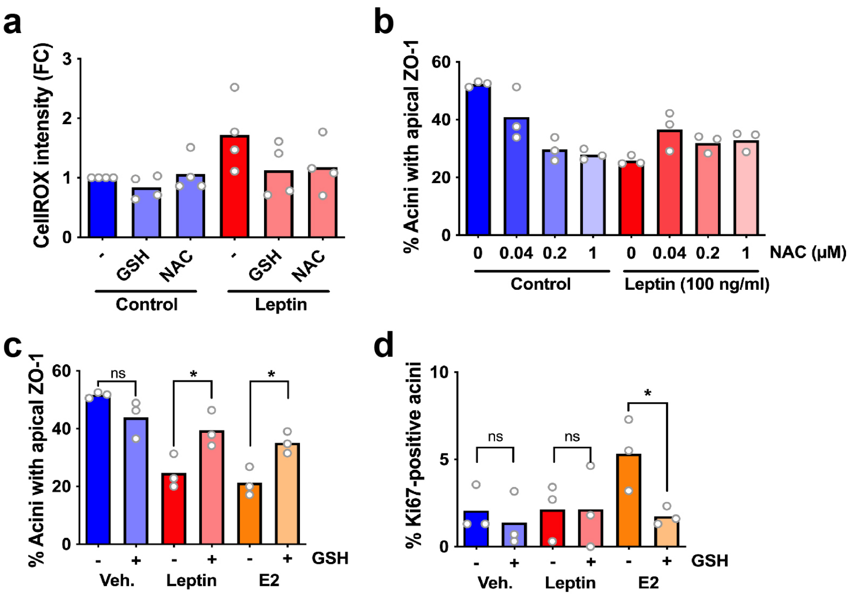

Supplementary Figure 7. ROS generation by leptin in breast acini. a ROS levels detected by the general ROS sensor CellROX in S1 cell acini treated for 30 min with leptin (100 ng/ml) or vehicle, in the absence or presence of glutathione (GSH; 0.16 mM) and N-acetylcysteine (NAC; 0.04 µM). **b** Proportion of acini with apical ZO-1 localization after treatment with leptin, in the presence or absence of NAC. **c-d** Apical ZO-1 localization (**c**) and expression of the Ki67 proliferation marker (**d**) in acini treated with vehicle, leptin (100 ng/ml), or β-estradiol (E2; 0.5 ng/ml), in the absence or presence of GSH (0.16 mM). *, $P < 0.05$ (unpaired t-test).
