# Supplementary material for: Reversion of breast epithelial polarity alterations caused by obesity
Source: NPJ Breast Cancer. 2023 May 9;9:35. doi: 10.1038/s41523-023-00539-w (PMC10170133; doi:10.1038/s41523-023-00539-w)
Supplement: Supplementary file 2 — Reporting Summary [file 41523_2023_539_MOESM2_ESM.pdf]

nature portfolio

Corresponding author(s): Pierre-Alexandre Vidi

Last updated by author(s): Jan 18, 2023

Reporting Summary

Nature Portfolio wishes to improve the reproducibility of the work that we publish. This form provides structure for consistency and transparency in reporting. For further information on Nature Portfolio policies, see our Editorial Policies and the Editorial Policy Checklist.

Statistics

For all statistical analyses, confirm that the following items are present in the figure legend, table legend, main text, or Methods section.

n/a	Confirmed	
☐	☒	The exact sample size (*n*) for each experimental group/condition, given as a discrete number and unit of measurement
☒	☐	A statement on whether measurements were taken from distinct samples or whether the same sample was measured repeatedly
☐	☒	The statistical test(s) used AND whether they are one- or two-sided<br>*Only common tests should be described solely by name; describe more complex techniques in the Methods section.*
☒	☐	A description of all covariates tested
☐	☒	A description of any assumptions or corrections, such as tests of normality and adjustment for multiple comparisons
☐	☒	A full description of the statistical parameters including central tendency (e.g. means) or other basic estimates (e.g. regression coefficient) AND variation (e.g. standard deviation) or associated estimates of uncertainty (e.g. confidence intervals)
☐	☒	For null hypothesis testing, the test statistic (e.g. *F*, *t*, *r*) with confidence intervals, effect sizes, degrees of freedom and *P* value noted<br>*Give P values as exact values whenever suitable.*
☒	☐	For Bayesian analysis, information on the choice of priors and Markov chain Monte Carlo settings
☒	☐	For hierarchical and complex designs, identification of the appropriate level for tests and full reporting of outcomes
☒	☐	Estimates of effect sizes (e.g. Cohen's *d*, Pearson's *r*), indicating how they were calculated

Our web collection on statistics for biologists contains articles on many of the points above.

Software and code

Policy information about availability of computer code

Data collection	The ZEN black software (Zeiss) was used for confocal data collection CellSens (Olumpus) was used for epifluorescence data collection.
Data analysis	FIJI (version 2.3.0) and QuPath (version 0.3.2), and published opensource custom MATLAB code (ref. provided) were used for image analyses. Graphpad Prism (version 9.5.0) was used to produce graphs and for statistical analyses.

For manuscripts utilizing custom algorithms or software that are central to the research but not yet described in published literature, software must be made available to editors and reviewers. We strongly encourage code deposition in a community repository (e.g. GitHub). See the Nature Portfolio guidelines for submitting code & software for further information.

Data

Policy information about availability of data

All manuscripts must include a data availability statement. This statement should provide the following information, where applicable:

- Accession codes, unique identifiers, or web links for publicly available datasets
- A description of any restrictions on data availability
- For clinical datasets or third party data, please ensure that the statement adheres to our policy

KTB donor information, available from the Virtual Komen Tissue Bank (https://virtualtissuebank.iu.edu), are provided in Suppl. Table S1. Adipokine measurements are listed in Suppl. Table S2. Imaging datasets from this study are available from the corresponding author on reasonable request.

Human research participants

Policy information about studies involving human research participants and Sex and Gender in Research.

Reporting on sex and gender	Findings in this paper, relevant to breast cancer, were derived from women cells/tissue samples and female mice.
Population characteristics	Human serum samples were from a biobank (Komen Tissue Bank, KTB) and from a previous study on diabetes prevention (HELP-PD). KTB donor characteristics are provided as supplementary information. HELP-PD participant characteristics are described in a publication that we reference.
Recruitment	No participant was recruited for this study.
Ethics oversight	Institutional Review Board, Wake Forest School of Medicine.

Note that full information on the approval of the study protocol must also be provided in the manuscript.

Field-specific reporting

Please select the one below that is the best fit for your research. If you are not sure, read the appropriate sections before making your selection.

☒ Life sciences ☐ Behavioural & social sciences ☐ Ecological, evolutionary & environmental sciences

For a reference copy of the document with all sections, see nature.com/documents/nr-reporting-summary-flat.pdf

Life sciences study design

All studies must disclose on these points even when the disclosure is negative.

Sample size	No sample size calculation was done. Sample sizes were decided based on feasibility of the analyses.
Data exclusions	For the analysis of adipose tissue (Fig 1b), 3 donors were excluded due to the absence of adipose tissue. For the analyses of polarity markers in tissues, donor or mice tissues with very few (<5) epithelial structures were excluded.
Replication	For each mouse or human tissue sample, each immunostaining was performed once but multiple epithelial structures from multiple regions of the tissue sections were evaluated. Analysis by ELISA of serum samples was done by RayBiotech. One serum sample was analyzed in quadruplicates. For cell-based assays, independent biological replicates were analyzed. The number of biological replicates is indicated in the figures (symbols on graphs).
Randomization	Mice were randomly assigned to the diet groups. For the Komen Tissue Bank breast and matching serum samples, donors were selected in 3 BMI categories by statisticians at the KTB, such as to balance age and race. Specific confounders were avoided, as described in the methods section.
Blinding	Human breast tissue samples were labeled with the tissue donor barcodes (Komen Tissue Bank) and mice were given a numerical identifier enabling blind image quantification. Visual scoring of cell culture assays was not blinded.

Reporting for specific materials, systems and methods

We require information from authors about some types of materials, experimental systems and methods used in many studies. Here, indicate whether each material, system or method listed is relevant to your study. If you are not sure if a list item applies to your research, read the appropriate section before selecting a response.

Materials & experimental systems

n/a	Involved in the study	
☐	☒	Antibodies
☐	☒	Eukaryotic cell lines
☒	☐	Palaeontology and archaeology
☐	☒	Animals and other organisms
☒	☐	Clinical data
☒	☐	Dual use research of concern

Methods

n/a	Involved in the study	
☒	☐	ChIP-seq
☒	☐	Flow cytometry
☒	☐	MRI-based neuroimaging

Antibodies

Antibodies used	All antibody used for this study are indicated in the Methods section, with supplier, catalog number, and dilution used.
Validation	All antibodies used in this study were validated for the specific application by the manufacturer.

Eukaryotic cell lines

Policy information about cell lines and Sex and Gender in Research

Cell line source(s)	HMT3522-S1 cells: immortalized non-neoplasmic human breast epithelial cells (Mina Bissell laboratory, BNL)<br>184B5: immortalized non-neoplasmic human breast epithelial cells (Cell Engineering Shared Resource of WFUHS)
Authentication	The cell lines were not authenticated but obtained directly from the source laboratories.
Mycoplasma contamination	All cell lines tested negative for mycoplasma
Commonly misidentified lines<br>(See ICLAC register)	None.

Animals and other research organisms

Policy information about studies involving animals; ARRIVE guidelines recommended for reporting animal research, and Sex and Gender in Research

Laboratory animals	C57BL/6 mice
Wild animals	NA
Reporting on sex	only female mice were used for this research using mammary glands.
Field-collected samples	NA
Ethics oversight	Animal Care and Use Committee of the Wake Forest School of Medicine (IACUC protocol #A18-136)

Note that full information on the approval of the study protocol must also be provided in the manuscript.
